# Cd47 deficiency in mouse brain under excitotoxic stress

**DOI:** 10.1371/journal.pcbi.1014897

**Published:** 2026-10-29

**Authors:** Mehrdad Kiamari, Amalia Perna, Matteo Santoro, Abdel El Ariss, Eloise Berson, Jing Zhao, Chandresh R. Gajera, Edward J. Fox, Kathleen S. Montine, Nima Aghaeepour, Thomas J. Montine

**Affiliations:** 1 Department of Pathology, Stanford University School of Medicine, Stanford, California, United States of America; 2 Department of Anesthesia, Perioperative and Pain Medicine, Stanford University School of Medicine, Stanford, California, United States of America; 3 BIO-Centre for Computational Biology, Mines Paris, PSL Research University, Paris, France; 4 Institut Curie, PSL Research University, Paris, France; 5 INSERM, Paris, France; North Carolina State University, UNITED STATES OF AMERICA

## Abstract

CD47, a transmembrane protein with well-established immune and neurodevelopmental roles, was recently identified as an abundant presynaptic protein in humans, non-human primates, and mice. To investigate how *Cd47* influences neuronal stress and injury, we performed single-nucleus RNA sequencing of hippocampi from wild-type (WT) and *Cd47* knockout (KO) mice exposed to kainic acid (KA) at convulsive (KA25; 25 mg/kg) or subconvulsive (KA3; 3 mg/kg) doses. Integrating cell-type annotations with Weighted Gene Co-expression Network Analysis (WGCNA) revealed distinct neuronal and glial gene modules whose co-expression structure differed by genotype and by KA exposure. Notably, certain modules showed KA-associated changes in co-expression structure only in *Cd47*-deficient cells. Enrichment analyses of these modules highlighted translational, bioenergetic, and synaptic gene sets. Comparing transcriptome-wide distributions using Wasserstein distance demonstrated that vehicle-treated KO cells most closely resembled WT cells exposed to KA3, of all fifteen pairwise comparisons. These findings suggest that *Cd47* loss shifts the hippocampal transcriptome toward a state resembling mild excitotoxic stress, implicating *Cd47* as a candidate regulator of neuronal stress adaptation and providing a hypothesis for testing that role.

## Author summary

Neurons in the brain must constantly adapt to stress, and understanding what molecular factors govern this adaptation is relevant to therapies for neurological diseases. In this study, we investigated the role of *Cd47*—a gene encoding a protein found abundantly at neuronal synapses—in how brain cells respond to excitotoxic injury, a form of neuronal stress implicated in epilepsy, stroke, and neurodegeneration. We performed single-nucleus RNA sequencing on hippocampal tissue from wild-type and *Cd47* knockout mice exposed to subconvulsive (KA3) and convulsive (KA25) doses of kainic acid, a well-established model of excitotoxic stress. To analyze the resulting data, we applied a multi-scale computational framework combining differential gene expression analysis, gene co-expression network analysis (WGCNA), and whole-transcriptome distribution comparisons using Wasserstein distance. Our analyses revealed that removing *Cd47* shifts the hippocampal transcriptome, under otherwise normal conditions, toward a state resembling subconvulsive excitotoxic stress. We also identified three genes—*Fth1*, *Rplp1*, and *Apoe*—as the most highly connected within the gene networks most affected by *Cd47* loss, making them candidates for future functional testing. These findings are consistent with a role for *Cd47* in setting the threshold at which hippocampal neurons mount a stress response; establishing that role will require direct functional testing, which this study does not provide. This work advances our understanding of neuronal vulnerability and offers a resource for research into disorders driven by excitotoxicity.

## Introduction

Neuronal stress responses are fundamental adaptive mechanisms that enable neurons to withstand a range of insults, including hyperexcitability stress, oxidative stress, immune response, and hypoxia [1,2]. Proper regulation of neuronal stress response is critical for maintaining brain health because when overwhelmed or dysregulated it can culminate in neuron damage, such as from excitotoxicity, and is proposed to contribute to neurodegenerative disorders such as Alzheimer’s disease, Parkinson’s disease, and amyotrophic lateral sclerosis [3,4]. A central goal in translational neuroscience is to understand the molecular regulators that govern neuronal stress response. *Cd47*, also known as integrin-associated protein (IAP), is a ubiquitously expressed transmembrane glycoprotein best known in the innate immune system where it acts as a “don’t eat me” signal; by binding to its receptor, signal regulatory protein-alpha (SIRPα) that is predominantly expressed on myeloid cells like macrophages and microglia, *Cd47* initiates an inhibitory signaling cascade that suppresses phagocytosis of healthy host cells [5–7].

A growing body of evidence also indicates that *Cd47* is integral in central nervous system (CNS) function. *Cd47* is abundantly expressed on presynaptic elements [8] and is expressed by astrocytes and oligodendrocytes in mice [9,10]. *Cd47* contributes to neurite outgrowth and neuronal development, and supports synapse protection from microglia-mediated elimination [8]. During development, activity-dependent enrichment of *Cd47* at synapses engages microglial SIRPα receptors to counteract complement-mediated phagocytosis [8]. *Cd47* expression is altered in several neurological conditions. For instance, neuronal CD47–SIRPα signaling acts as an inhibitory checkpoint for microglial phagocytic activity at synapses, linking CD47 signaling to microglia-mediated remodeling and inflammatory responses in the CNS [8,11]. Furthermore, in experimental models of cerebral ischemia, *Cd47* deficiency reduced infarct volume and dampened post-ischemic inflammation [12].

Despite these important findings, the cell-intrinsic role of *Cd47* in orchestrating neuronal responses to stress remains incompletely understood. It is particularly unclear how the absence of *Cd47* alters the transcriptional landscape of neurons and their subsequent vulnerability to direct neuron stressors in vivo. Addressing this knowledge gap is critical for determining whether *Cd47* signaling represents a viable therapeutic target for conditions characterized by neuronal stress and injury. In this study, we tested the hypothesis that *Cd47* is a key regulator of neuron response to hyperexcitability stress and excitotoxicity. We employed subconvulsive (low) and convulsive (high) doses of kainic acid (KA) to cause hyperexcitability stress in mice, recapitulating key pathophysiologic features observed in epilepsy and chronic neurodegeneration [13,14]. To dissect the transcriptional consequences of *Cd47* deficiency under excitotoxic stress, we designed a multi-scale analytical framework (Fig 1). We combined differential expression analysis to pinpoint individual gene targets, global transcriptomic comparisons to assess large-scale shifts, and Weighted Gene Co-expression Network Analysis (WGCNA)—a method widely used in neuroscience [15,16]— to identify hub genes within neuronal and glial subpopulations. This integrated approach provides a systems-level, cell-type-resolved description of the hippocampal transcriptome in the presence and absence of *Cd47* under graded excitotoxic stress. It is designed to generate hypotheses about how *Cd47* modulates neuronal vulnerability rather than to test them mechanistically.

**Fig 1:**
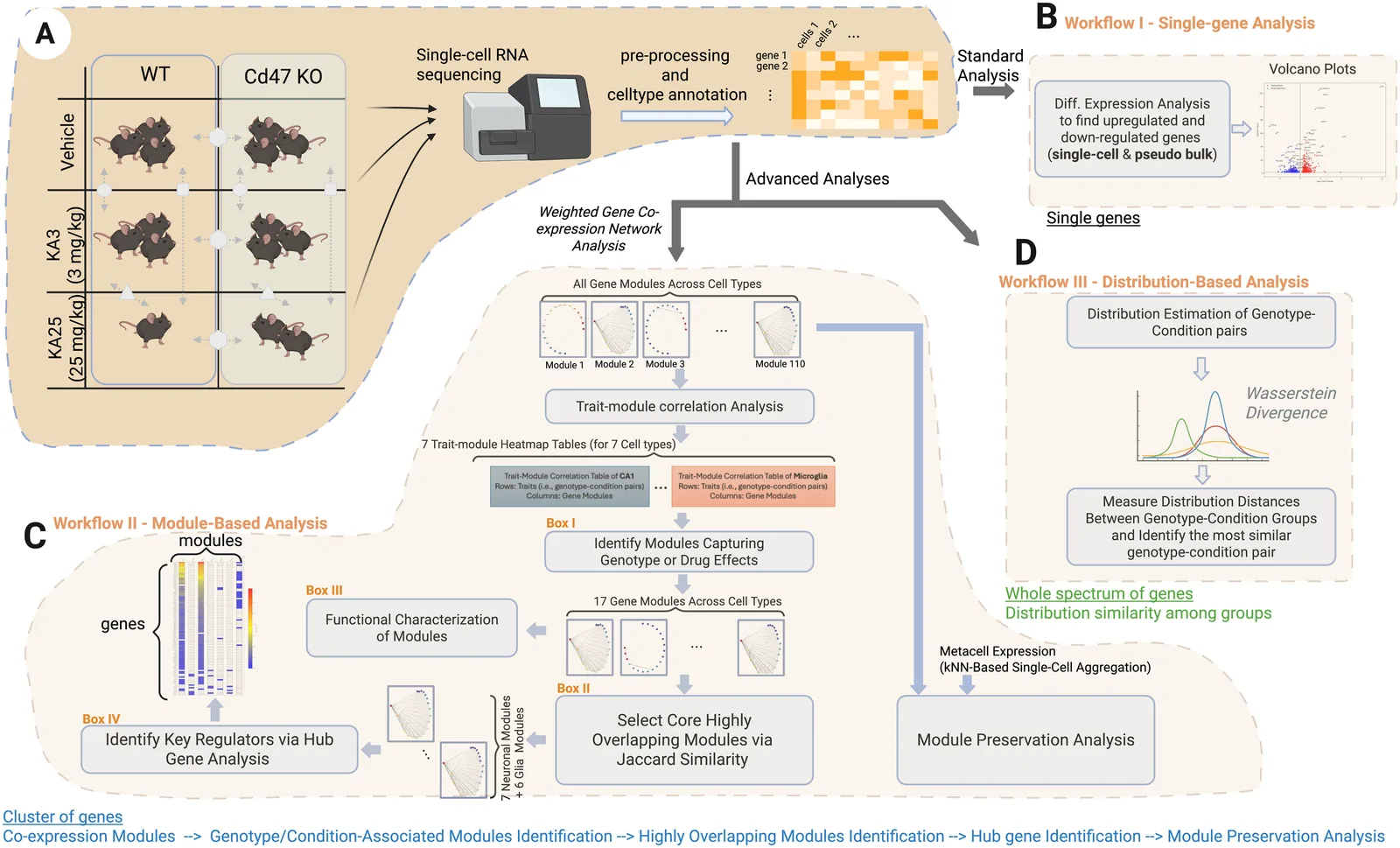
Overview of experimental design and data analysis approaches. Approaches 1–3 provide complementary perspectives, enabling the identification of emergent network properties and interactions beyond standard differential expression analyses. **A)** The experimental cohort consisted of 7 wild-type (WT) and 8 *Cd47* knockout (KO) mice (8 male and 7 female in total; per-group sex composition in Methods) treated with saline 0.9% (Veh; n = 3 WT and n = 3 KO), low-dose kainic acid (KA3, 3 mg/kg; n = 3 WT and n = 3 KO), or high-dose kainic acid (KA25, 25 mg/kg; n = 1 WT and n = 2 KO). In the schematic, geometric symbols (circles, triangles, squares, and hexagons) indicate the comparison type, distinguishing contrasts performed either across treatments within the same genotype or across genotypes within the same treatment.Single-nucleus RNA sequencing was performed on hippocampus; KA doses were validated to be subconvulsive and convulsive (Fig O in S1 Appendix). All approaches were performed across all identified cell types. **B)** Approach 1 – Single-gene analysis (Differential Expression Analysis): identifying up- and down-regulated genes between WT and KO under different conditions, performed both at the single-nucleus level and on pseudobulk profiles, with volcano plots shown for each. **C)** Approach 2 – Gene-set (module) analysis (Network and Module-Based Analysis): constructing cell type–specific gene co-expression networks using Weighted Gene Co-expression Network Analysis (WGCNA) to group genes into modules of highly correlated expression. correlation analysis of module eigengenes with traits (i.e., genotype-condition pairs) identified modules capturing genotype- or drug-associated effects. Subsequently, to identify highly overlapping core modules across cell types, the modules were compared using Jaccard similarity. Within these core modules, genes are ranked by network centrality measures (e.g., intramodular connectivity, degree, or betweenness) to highlight hub genes likely to play key regulatory roles across neuronal cell types. Finally, enrichment analyses are performed to functionally characterize core modules and interpret their biological relevance in the context of genotype or drug response. Module robustness is assessed by re-evaluating the constructed modules in metacells (PCA followed by k-nearest-neighbour aggregation within each mouse and cell type), scored by a permutation-calibrated *Z*_summary_ statistic combining density and connectivity. **D)** Approach 3 – Whole-transcriptome analysis (Distribution-Based Similarity Analysis): estimating the probability distribution of each genotype–condition pair, then measuring pairwise distances between these distributions to determine which genotype–condition combinations are most similar in their overall transcriptional profiles. Approaches 1–3 are applied to the same single-nucleus dataset and are therefore not statistically independent; their agreement indicates stability across analytical treatments rather than independent replication. Created in BioRender. Kiamari, **M.** (2026) https://core.local.biorender.dev/api/short-link/jgqyvkw.

## Results

### Overview of single-nucleus transcriptomic data and analytical approaches

We profiled hippocampal single-nucleus transcriptomes from WT and *Cd47* KO mice exposed to vehicle or KA at subconvulsive (KA3, 3 mg/kg) or convulsive (KA25, 25 mg/kg) doses (Fig 1A; Veh, n = 3 per genotype; KA3, n = 3 per genotype; KA25, n = 1 WT and n = 2 KO; see Methods) to capture genotype- and stress-associated transcriptional differences. Analyses integrated single-gene-level (Fig 1B), module-level (Fig 1C) and whole-transcriptomic (Fig 1D) frameworks to reveal complementary aspects of *Cd47*-dependent hippocampal response to lower vs. higher levels of excitotoxic stress across cell types.

Our analyses operate at three levels of resolution, and we state the unit of replication for each. Single-gene contrasts reported as significant are tested at the level of the mouse using pseudobulk profiles (Fig F in S1 Appendix). The nucleus-level volcano plots in Fig 2 and Figs A–E in S1 Appendix display the full landscape of variation and are used for gene nomination. Co-expression networks were constructed at the level of the nucleus, because a within-cell-type correlation network estimated from 15 samples would be underdetermined; the resulting modules were then re-evaluated in a metacell representation of the same data to test whether their co-expression structure is retained (Table D in S1 Appendix), and module eigengenes were additionally analysed at the level of the mouse with linear models including genotype, treatment and their interaction.

**Fig 2:**
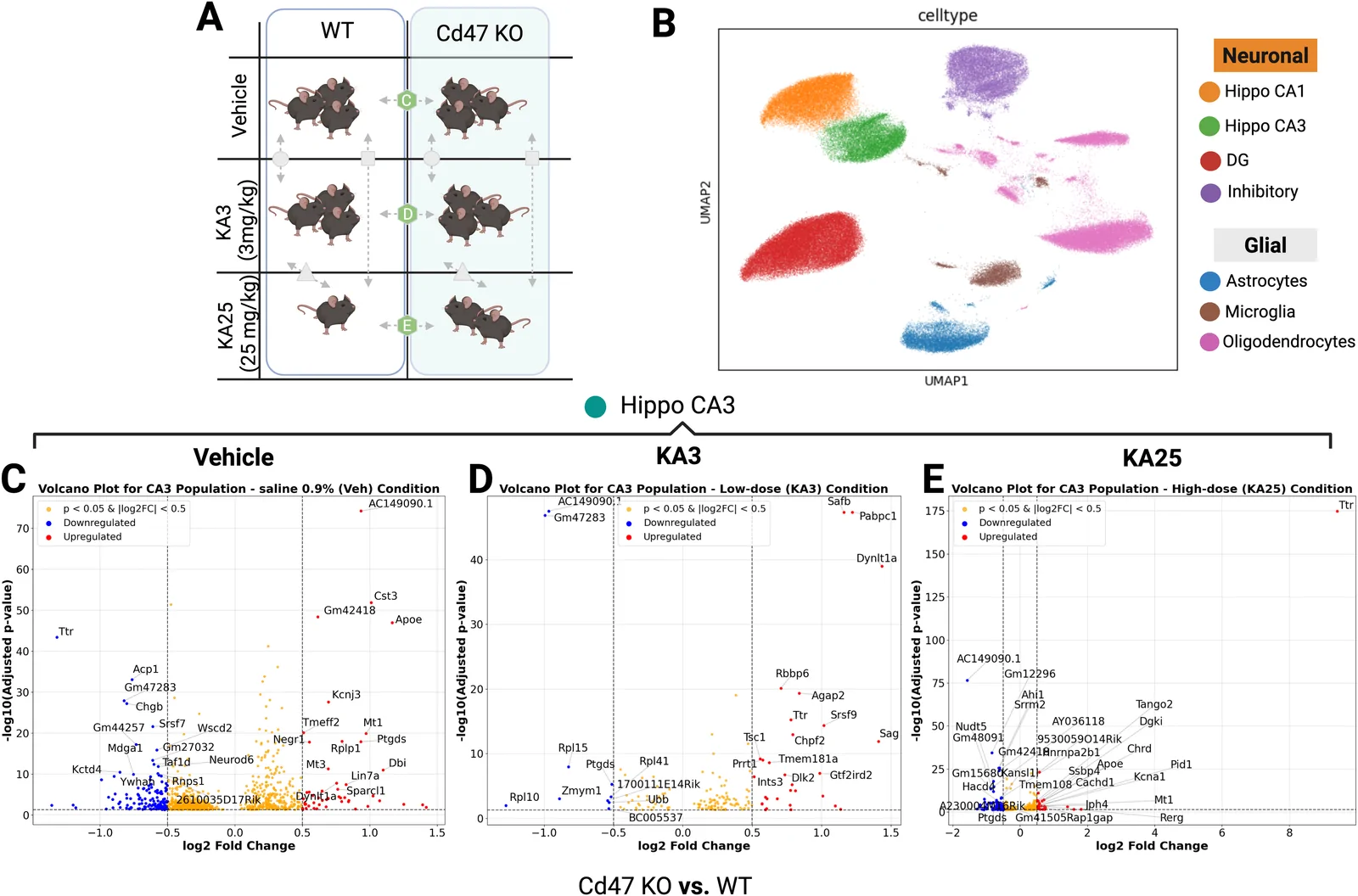
Hippocampal transcriptional landscape of *Cd47* KO mice at baseline and following excitotoxic stress. **(A) Exposure:** Mice (WT or *Cd47* KO) received one of three intraperitoneal injections: vehicle (0.9% saline) or two different levels of excitotoxic stress: subconvulsive kainic acid (KA3; 3 mg/kg) or convulsive kainic acid (KA25; 25 mg/kg). **(B) Cell type annotation:** Cell clusters were identified using principal component analysis (PCA) for dimensionality reduction followed by Leiden clustering based on transcriptional similarity. Biological identities were then assigned using differential expression analysis to identify cluster-enriched marker genes. Canonical markers and reference-guided annotation resolved major neuronal and glial populations, establishing a cell type–resolved framework. **(C-E) CA3 condition-dependent Differentially Expressed Genes (DEGs):** Volcano plots illustrate the gene expression changes within CA3 pyramidal neurons, which showed the most nucleus-level differences in our analysis comparing *Cd47* KO against WT mice under the three different conditions. See Figs A–E in S1 Appendix for volcano plots of the other cell types, and response to kainic acid. Panels C–E show single-nucleus analysis and are used to nominate candidate genes; nuclei from the same animal are not independent biological replicates, and the corresponding tests at the level of the mouse are shown in Fig F in S1 Appendix. Panel E (KA25) is descriptive because it includes one WT and two KO animals; therefore, no mouse-level contrast at this dose can reach conventional significance. The x-axis shows the log2 fold change, and the y-axis represents statistical significance ($-\log_{10}(\text{adjusted p-value})$). Genes that are significantly upregulated (red) or downregulated (blue) in the *Cd47* KO mice compared to WT are highlighted. Orange points denote genes that are significant but show small change (absolute log2 fold change < 0.5). The top 15 upregulated and top 15 downregulated genes (by adjusted p-value) are labeled in each plot. Created in BioRender. Kiamari, M. (2026) https://core.local.biorender.dev/api/short-link/kvktzf0.

### *Cd47* KO alters the transcriptional landscape of excitotoxic stress in a context-dependent manner (Approach 1)

Transcriptional differences between *Cd47* KO and WT mice were strongly context dependent, varying with the level of excitotoxic stress induced by KA. Following varying KA exposure (Fig 2A) and single-nucleus transcriptomic analysis, unbiased Leiden clustering of the pooled nuclei yielded transcriptionally distinct clusters whose cluster-enriched marker genes were compared to the Allen Institute’s mouse brain atlas [17] and to canonical markers (see Methods) to annotate seven major neuronal (CA1, CA3, dentate gyrus (DG), inhibitory) and glial (astrocytes, oligodendrocytes, and microglia) cell types (Fig 2B). These seven populations emerged from unbiased clustering and were not specified a priori. Clusters that could not be confidently assigned to one of these populations, and clusters containing too few nuclei per genotype–treatment group to support within-cell-type network construction, were not carried forward; because excitotoxic stress is likely to affect these populations as well, their responses are not captured here. Nuclei counts for every mouse and every analysed cell type are given in Tables A1-A5 in S1 Appendix. Relatively up-regulated and down-regulated genes under varying KA exposures for each cell type were identified by volcano plots. CA3 pyramidal neurons (Fig 2C-2E) showed the most nucleus-level differences (see Figs A–C in S1 Appendix for other cell types and Figs D-E in S1 Appendix for the response to KA within each genotype). Our CA3 finding is consistent with morphologic data showing that mouse CA3 hippocampal pyramidal neurons are among the most vulnerable to KA [18]. At baseline (vehicle treatment), CA3 nuclei from *Cd47* KO mice showed higher expression than WT of the vesicular glutamate transporter *Slc17a7* and of a large set of cytoplasmic ribosomal protein and oxidative-phosphorylation subunit genes, including *Rps28* and *Rpl27*. We describe this as a coordinated shift in the abundance of translational and bioenergetic transcripts rather than as a change in neuronal identity. Transcripts of this class are also sensitive to the number of nuclei recovered per animal, which differed between genotypes in the vehicle arm (Tables A1-A5 in S1 Appendix); all mouse-level testing was therefore performed after library-size normalisation (Fig F in S1 Appendix), and this gene set is reported as a candidate difference rather than as an established one. Genes with roles at the synapse were altered in both directions, and we group them by direction of change rather than by function, because they span transcriptional regulation, cell adhesion, ion-channel modulation and vesicular secretion, and a single functional label would misrepresent them. Higher in *Cd47* KO were *Kcnj3* (GIRK1, a G-protein-gated inwardly rectifying K^+^ channel subunit mediating slow inhibitory postsynaptic currents [19]), *Negr1* (an IgLON-family GPI-anchored adhesion molecule regulating neurite outgrowth and synapse number [20]) and *Tmeff2* (a transmembrane protein with EGF- and follistatin-like domains expressed in forebrain neurons [21]). Lower in *Cd47* KO were *Neurod6* (a bHLH transcription factor of the excitatory neuron lineage that also confers tolerance to oxidative stress [22]), *Mdga1* (a GPI-anchored adhesion molecule that binds neuroligin-2 and constrains inhibitory synapse formation [23]), *Kctd4* (a potassium-channel tetramerisation-domain protein; several KCTD-family members act as auxiliary subunits of GABA_B_ receptors [24]), *Wscd2* (a neuronally expressed WSC-domain protein of largely uncharacterised function), *Chgb* (chromogranin B, a granin precursor of the regulated secretory pathway [25]) and *Ttr* (transthyretin, whose loss reduces susceptibility to AMPA-induced neurodegeneration [26]).

Under KA exposure the genotype contrast shifted toward genes governing synaptic transmission and plasticity, and stress response. At both KA doses, the most prominent nucleus-level differences emerged in greater expression levels of Tsc1 (negative regulator of the mTOR pathway) [27], Prrt1 (AMPA receptor trafficking) [28], Tmem108 (vesicle trafficking and receptor recycling at synapses) [29], Tango2, and Ttr (synaptic stress resilience) in WT than *Cd47* KO [26,30].

In the mouse-level analysis under KA3, no gene reached statistical significance in the genotype contrast in any cell type (Fig F in S1 Appendix). This is consistent with the whole-transcriptome ranking, in which *Cd47* KO-KA3 and WT-KA3 are among the most similar of the fifteen pairwise comparisons (Fig 7): the two genotypes differ little from each other once KA3 has been applied. The KA25 genotype contrast is presented as descriptive only: with one WT and two KO animals the exact permutation distribution contains only (31)=3 arrangements, so the smallest attainable *p*-value is 1/3≈0.333, before any multiple-testing correction, and no gene can reach conventional significance regardless of effect size.

In the vehicle condition, mouse-level pseudobulk testing recovered the central baseline observation. *Apoe*, *Rplp1* and *Fth1* — the three genes that the co-expression analysis below identifies as the most highly connected in the *Cd47*-associated neuronal modules — were higher in KO than WT in every cell type in which they were detected, with no cell type showing a discordant direction (Fig F in S1 Appendix). *Rplp1* ($\log_{2}$FC = 4.22, adjusted *p* = 0.046) and *Fth1* ($\log_{2}$FC = 3.92, adjusted *p* = 0.046) reached false-discovery-rate significance in astrocytes, and the remaining contrasts were nominally significant in the same direction (*Apoe*: CA3 $\log_{2}$FC = 4.53, *p* = 0.031; oligodendrocytes 3.70, *p* = 0.024; inhibitory neurons 4.50, *p* = 0.039; microglia 3.76, *p* = 0.018; DG 4.21, *p* = 0.038; CA1 4.27, *p* = 0.041). Because nuclei recovery differed substantially between genotypes in this arm (Tables A1-A5 in S1 Appendix), all mouse-level testing was performed after library-size normalisation, which removes the dependence of pseudobulk sums on the number of contributing nuclei.

### Identification and characterization of cell type-specific gene modules using WGCNA (Approach 2)

To move beyond single-gene changes and identify coordinated transcriptional programs, we applied WGCNA [31,32] across all genotype–treatment groups and cell types, yielding 110 cell type-specific modules (Fig 3A; module–trait correlation heatmaps shown in Figs G and H in S1 Appendix; module sizes for the genotype- and KA-associated modules examined below in Table B in S1 Appendix). These heatmaps summarize the correlation of each module eigengene with each genotype–treatment condition, providing a comprehensive view of how module–trait association shifts across genotypes or KA exposure states. We then distilled this global module–trait correlation framework into focused pairwise comparisons across genotype and treatment groups to identify the modules showing the strongest and most significant differential associations under each condition.

**Fig 3:**
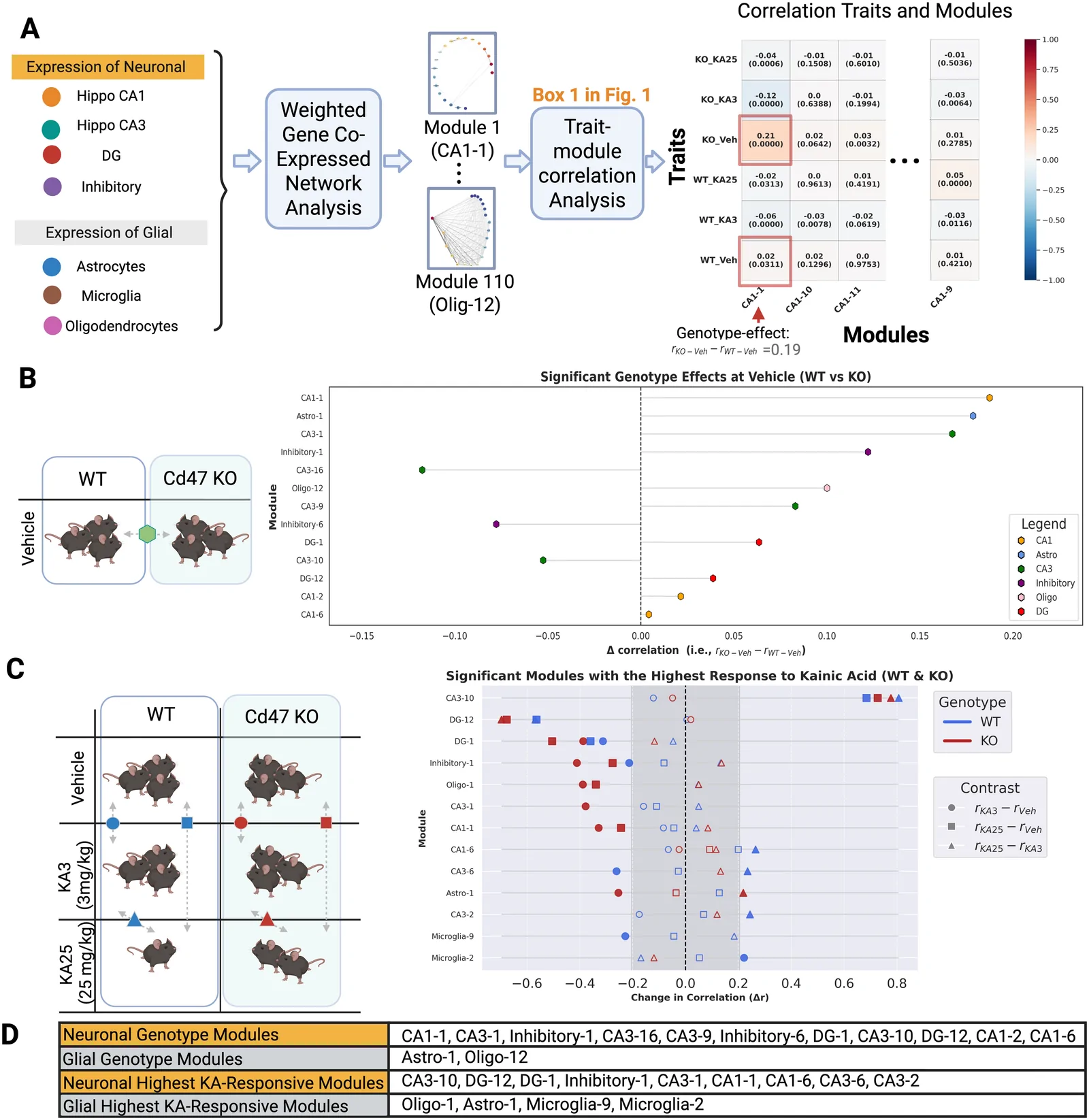
Functional association of identified gene co-expression networks (modules) is altered by genotype and treatment. Analysis is based on statistically significant (adjusted p-value < 0.05) changes in module-trait correlations at the nucleus level; mouse-level linear models on the same eigengenes are reported in the Results. Δr measures a change in the correlation between a module eigengene and a condition; it is a property of co-expression structure and does not by itself indicate a change in expression level (see Results). Schematic icons indicate the contrasts: hexagons for genotype comparisons (*Cd47* KO vs WT) under Veh conditions, circles for low-dose KA at 3 mg/kg vs Veh (KA3 vs Veh), squares for high-dose KA at 25 mg/kg vs Veh (KA25 vs Veh), and triangles for dose-escalation comparisons (KA25 vs KA3). In panel C, blue indicates WT and red indicates *Cd47* KO. **(A)** Schematic identifying genotype- and treatment-associated gene co-expression modules through WGCNA and module–trait correlation analysis. **(B)** Baseline genotype effect. Intrinsic impact of the *Cd47* knockout by ranking modules based on the difference in correlation between vehicle-treated KO and WT mice. Negative values indicate a weaker association of the module eigengene with the KO condition, while positive values indicate a stronger association. **(C)** Treatment-induced effects. Response to Kainic Acid (KA) by displaying modules with a substantial change in correlation. The plot shows the response to KA across three contrasts—low-dose (KA3 vs. Vehicle), high-dose (KA25 vs. Vehicle), and the dose-escalation effect (KA25 vs. KA3)—within both WT (blue) and KO (red) genotypes. Negligible changes are colored gray. Modules are sorted by response magnitude, with the strongest responders at the top. **(D)** Top genotype- and Highest KA-responsive modules. Modules identified in panels B and C are grouped by cell type and effect, including neuronal and glial modules associated with genotype at baseline and those exhibiting the strongest responses to KA exposure. Module preservation analysis, assessing whether the co-expression structure of each module is retained in a metacell-based representation of the same data, is reported for all tested modules in Table D in S1 Appendix. Created in BioRender. Kiamari, **M.** (2026) https://core.local.biorender.dev/api/short-link/f7z6yks.

Interpreting Δ correlation. Each module is summarised by its eigengene, the first principal component of the expression of its members across nuclei. Δr denotes the difference between two module–trait correlation coefficients and therefore measures how strongly a module eigengene tracks one condition relative to another. It is a property of the correlation structure among genes and is not equivalent to a change in mean expression, transcription rate, or cellular activity: a module can show a large Δr with no change in mean expression if its members become more or less tightly co-varying, and conversely. We therefore describe Δr in these terms throughout, and where an expression-level claim is made we test expression level directly (Fig F in S1 Appendix). Module eigengenes were additionally computed from the within-mouse metacell data, averaged within each animal, and analysed with linear models including genotype, treatment and their interaction, with Benjamini–Hochberg correction across modules within each cell type and model term (see Methods). This analysis was restricted to the vehicle and KA3 conditions (*n* = 12 mice), because the KA25 arm contains one WT and two KO animals and cannot support inference, and to the 85 modules found to be preserved. Three modules reach false-discovery-rate significance at the level of the mouse, all in inhibitory neurons and all for the treatment term (KA3 versus vehicle): Inhibitory-6 (β=−0.74, 95% CI −1.12 to −0.35, FDR = 0.015), Inhibitory-18 (β=−0.47, 95% CI −0.78 to −0.15, FDR = 0.023) and Inhibitory-22 (β=−0.49, 95% CI −0.83 to −0.15, FDR = 0.023), each showing a lower eigengene under KA3 than under vehicle. No genotype main effect and no genotype-by-treatment interaction survives correction in any cell type. At *n* = 12 the model is not powered to resolve genotype differences of the magnitude implied by the nucleus-level analysis, and the module-level results below are therefore reported as differences in co-expression structure rather than as demonstrated differences in expression level.

#### Modules whose co-expression structure differs by genotype.

We identified 13 modules with a significantly different module-trait correlation between vehicle-treated *Cd47* KO and WT mice (Fig 3B). This comparison isolates the intrinsic association of the *Cd47* knockout, where a positive change in correlation (Δr) indicates a stronger association of the module eigengene with the *Cd47* KO condition, and a negative value indicates a weaker association. Among neuronal modules, CA1–1 and CA3–1 — both with *Rplp1*, *Apoe* and *Fth1* as their most connected genes, and both enriched for cytoplasmic translation and oxidative phosphorylation — exhibited the greatest genotype difference, while among glial modules, Astro-1 showed the largest effect, indicating that co-expression structure in these core neural and glial networks differs between genotypes even under normal conditions.

#### Modules whose co-expression structure changes with kainic acid.

We detailed the network response to varying levels of excitotoxic stress by identifying modules with a substantial change in correlation (i.e., |Δr| to be greater than 0.2) following KA exposure (Fig 3C). This threshold was chosen to highlight modules with a change corresponding to at least a small-to-medium effect size, thereby focusing the analysis on biologically meaningful shifts. While Fig 3C displays the top responders, a complete visualization of all modules with a significant response to KA is provided in Fig I in S1 Appendix. This revealed both shared and genotype-specific patterns. For instance, when exposed to subconvulsive KA, both genotypes showed a conserved response involving an increase in the CA3–10 module–trait correlation (positive Δr) and a decrease in the DG-12 module–trait correlation (negative Δr). However, *Cd47* status clearly modulated the pattern, as the Astro-1 eigengene shifted its trait association in KO but not in WT mice, and microglial modules showed smaller Δr in KO than in WT mice. Likewise, marked negative Δr shifts—restricted to KO and absent in WT—occurred most prominently in modules CA1–1, CA3–1, and Astro-1. Under convulsive stress, these shared signatures were amplified, and dose-dependent divergences emerged. In summary, KA is associated with a conserved pattern of increased CA3–10 and decreased DG-12 module–trait correlation in both genotypes, while glial and CA1 modules show genotype-dependent Δr.

#### Identifying highly overlapping modules via jaccard similarity.

As seen in Fig 3D, there were several modules associated with genotype and KA exposure. We determined the degree of overlap among these modules using the Jaccard index. In Figs 4A (neuronal) and 4B (glial), each module is represented as a node and edges denote shared gene membership quantified by the Jaccard index, which ranges from 0 (no shared genes) to 1 (identical gene composition). To emphasize the most meaningful relationships, only pairs with Jaccard≥0.2 (top 20%) are shown, while Figs 4C and 4D illustrate the corresponding statistical significance using bubble plots where bubble size reflects overlap magnitude and color encodes $-\log_{10}(\text{adjusted p-value})$. This integrated view revealed that seven neuronal module pairs were both highly overlapping and significantly enriched ($-\log_{10}(\text{adjusted p-value})>1.3$). Among neuronal modules, CA1–1 and CA3–1 show the strongest and most significant overlap (Jaccard = 0.47); both modules independently return cytoplasmic ribosome and oxidative phosphorylation terms in their own enrichment analyses (Fig 5A, 5B), so we describe the overlap as a translational–bioenergetic gene set common to both pyramidal populations, whereas Astro-1 and Microglia-9 share a substantial fraction of their gene membership, but we attach no functional claim to that overlap: Microglia-9 returns ribosomal and mitochondrial terms (Fig 5F), Astro-1 did not yield significant enrichment, and a high Jaccard index measures shared membership rather than shared function. Most module pairs (≈85%) had *Jaccard* < 0.2 and are omitted for clarity; a full comparison using a less stringent cutoff (Jaccard≥0.05) is provided in Fig J in S1 Appendix.

**Fig 4:**
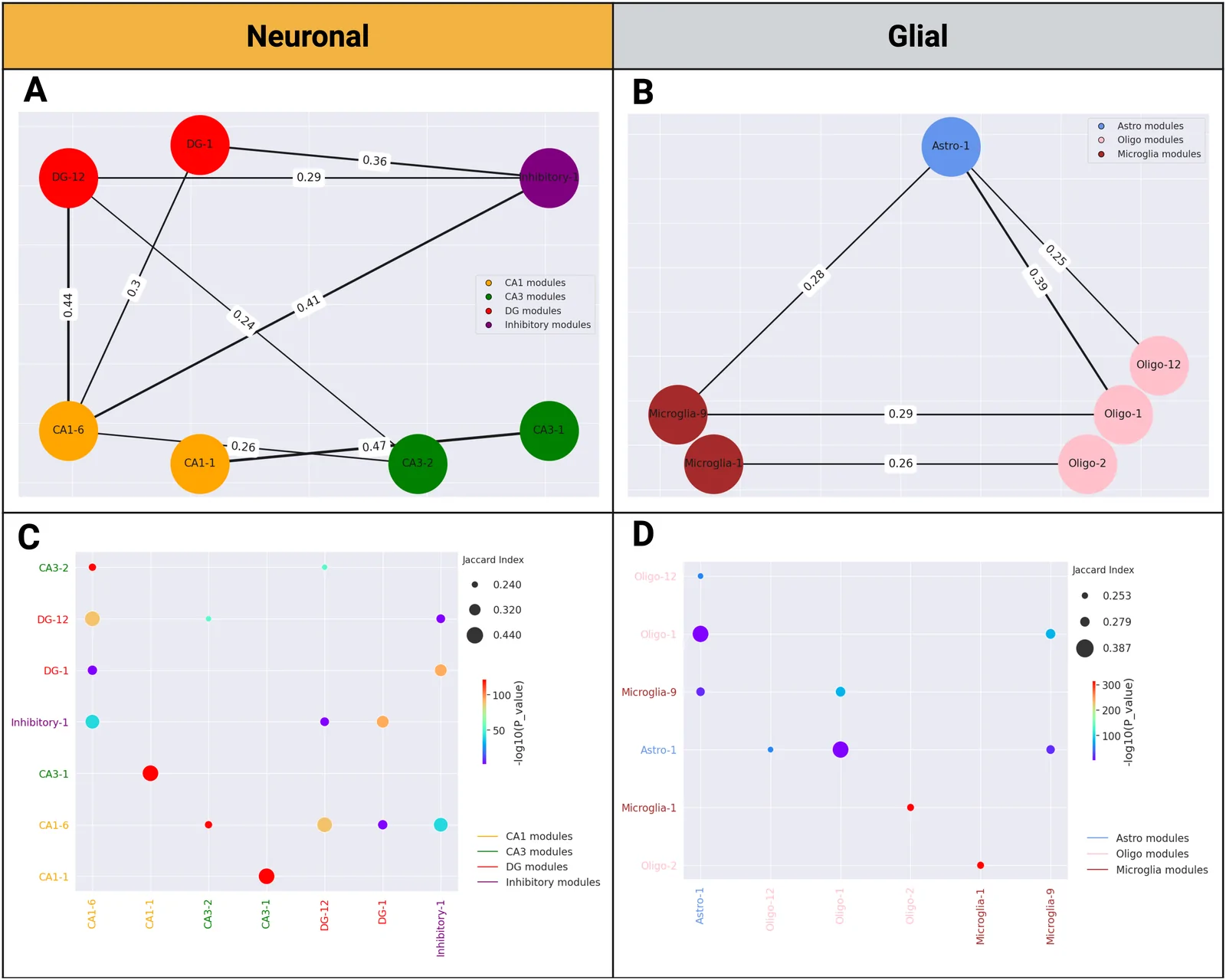
Cross-module analysis identifies highly overlapping (i.e., Jaccard similarity score ≥0.2) genotype- or drug-responsive modules. This figure visualizes the overlap between modules shown in Fig 3D. The analysis is filtered to display only pairs considered both highly overlapping (Jaccard similarity score ≥0.2) and statistically significant ($-\log_{10}(\text{adjusted p-value})>1.3$). **(Panels A-B)** Measuring Jaccard Similarity of Modules: The network diagrams illustrate the interconnectivity between modules for neuronal **(A)** and glial **(B)** cell types. Each node represents a module, and the connecting edges are labeled with the Jaccard similarity score, indicating the proportion of shared genes. **(Panels C-D)** Statistical Significance via Bubble Plot: The bubble plots provide statistical context for the overlaps in neuronal **(C)** and glial **(D)** modules. The size of each bubble corresponds to the Jaccard similarity score (magnitude of overlap), while its color reflects the statistical significance ($-\log_{10}(\text{adjusted p-value})>1.3$). All colored bubbles denote statistically significant intersections, highlighting the most biologically relevant module relationships.

**Fig 5:**
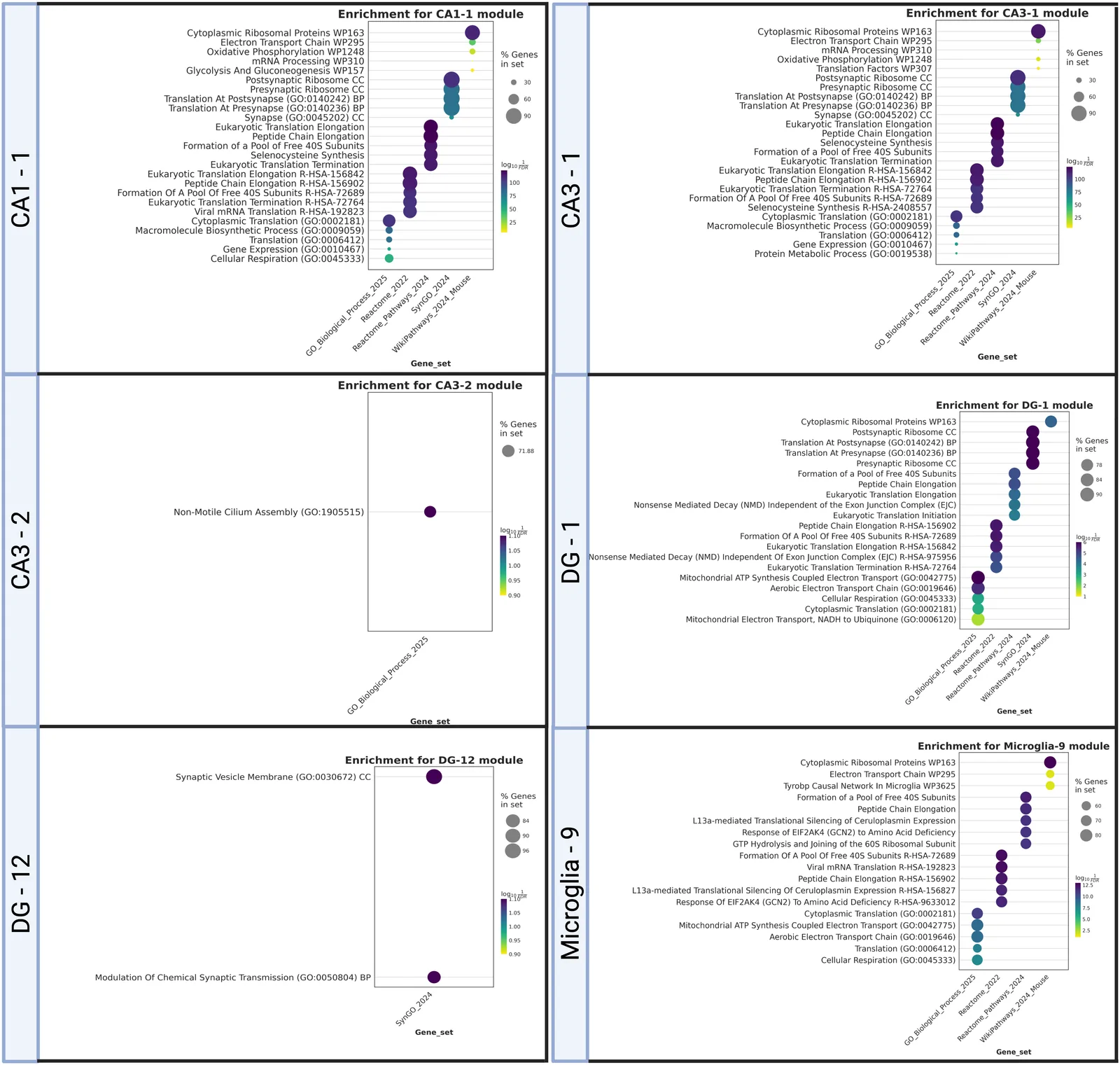
Functional enrichment of highly overlapping genotype- or drug-responsive modules. Highly overlapping genotype- or drug-responsive modules identified a bioenergetic-translational network associated with synaptic activity and microglial engagement (CA1-1, CA3-1 and Microglia-9), KA-associated disruption of DG synaptic modules (DG-1 and DG-12), and a *Cd47*-dependent difference in the CA3-2 primary cilium module. To define the biological functions of CA1-1 **(A)**, CA3-1 **(B)**, CA3-2 **(C)**, DG-1 **(D)**, DG-12 **(E)** and Microglia-9 **(F)**, functional enrichment analysis was performed using Gene Ontology (GO), KEGG, WikiPathways, REACTOME and SynGO. Circle size reflects the proportion of module genes associated with each term, and color indicates statistical significance as the negative log-transformed false discovery rate, with warmer colors indicating greater significance.

We performed comprehensive functional enrichment analysis on the genotype- or KA-associated modules identified in Fig 3D by testing each module’s gene set for overrepresented biological processes and pathways, thereby defining the dominant functions represented within these *Cd47*-relevant networks (Fig 5, Figs K–M in S1 Appendix). Fig 5 showed all highly overlapping genotype- or KA-responsive modules that yielded significant enrichment across the pathway databases used, including CA1–1, CA3–1, CA3–2, DG-1, DG-12, and Microglia-9; the remaining highly overlapping genotype- or treatment-responsive modules did not yield significant enrichment.

This analysis revealed three themes. First, a translational–bioenergetic gene set links transcripts encoding the protein synthesis machinery with those encoding oxidative phosphorylation components, a theme prominent across key neuronal and glial modules (Figs 5A, 5B, 5F). The highly similar neuronal modules CA1–1 and CA3–1 were strongly enriched for both oxidative phosphorylation and cytoplasmic ribosome terms. This same theme was mirrored in Microglia-9. We treat the enrichment as descriptive of module composition rather than as evidence of altered metabolic or translational activity.

Second, our analysis uncovered a genotype-associated ciliary gene set (Fig 5C). The CA3–2 module, which is enriched for genes involved in “non-motile cilium assembly,” shifted its eigengene association with treatment in WT but not in *Cd47* KO mice. Primary cilia concentrate receptors for Hedgehog, Wnt, and other signaling pathways on neurons, and ciliary gene expression is dynamically regulated in several injury models [33,34]. This raises the testable hypothesis that CD47 is required for a ciliary transcriptional response to excitotoxic challenge. We emphasise that this is a hypothesis generated by the co-expression analysis. We did not image cilia, quantify ciliary protein, or manipulate ciliary signalling, and the observation rests on a single module in a single cell type. Direct tests would include immunostaining for ARL13B or AC3 in CA3 after KA, and conditional disruption of ciliogenesis on the *Cd47* KO background.

Third, modules enriched for transcripts encoding synaptic and neurotransmitter-release proteins, such as DG-1 and DG-12, showed the largest Δr under KA25 treatment (Figs 5D, 5E), indicating that co-expression among these transcripts is least stable under the convulsive dose.

#### Co-expression hub analysis identifies highly connected genes within *Cd47*-sensitive modules.

Throughout, hub denotes a co-expression hub — a gene with high degree centrality in the weighted correlation network — and not a transcriptional regulatory hub or a protein–protein interaction hub. High co-expression centrality indicates that the expression of a gene co-varies with many other module members across nuclei; it does not imply that the gene regulates those genes, and for a secreted protein such as ApoE no direct regulatory role is implied.

To identify the most highly connected genes, we performed a co-expression hub analysis for neuronal modules shown in Fig 4A. By quantifying intramodular gene connectivity using degree centrality, this analysis produced a ranked list of the most highly connected genes within each module. Modules CA1–1 and CA3–1—which we previously identified as being significantly altered by the *Cd47* knockout (Fig 3B)— were particularly notable, exhibiting centrality scores nearly ten-fold higher than other neuronal modules, indicating exceptionally strong internal co-expression. Their co-expression subnetworks for the top 20 most connected genes are shown in Figs 6A and 6B, with Fig N in S1 Appendix showing other modules. Both modules were among the most strongly preserved in the dataset when their network structure was re-evaluated using within-mouse metacells (*Z*_summary_ = 35.85 for CA1–1 and *Z*_summary_ = 25.58 for CA3–1; Table D in S1 Appendix), supporting the robustness of the network topology used for hub-gene identification. Fig 6C further ranks the genes with consistently high centrality across multiple neuronal populations, focusing on those shown in Fig 3D. The top genes from this ranking are *Fth1*, *Rplp1*, and *Apoe*, whose annotated functions fall into three areas—iron homeostasis and oxidative defense (*Fth1*), translational control and stress-adaptive protein synthesis (*Rplp1*), and lipid transport and neuron–glia repair signaling (*Apoe*). High co-expression centrality does not establish that these genes are functionally important, that they regulate the modules in which they sit, or that they sustain resilience under KA-induced stress. We present them as candidates for functional follow-up. Establishing such a role would require cell-type-specific knockdown or overexpression on the *Cd47* KO background followed by measurement of neuronal survival after KA, which is outside the scope of this study.

**Fig 6:**
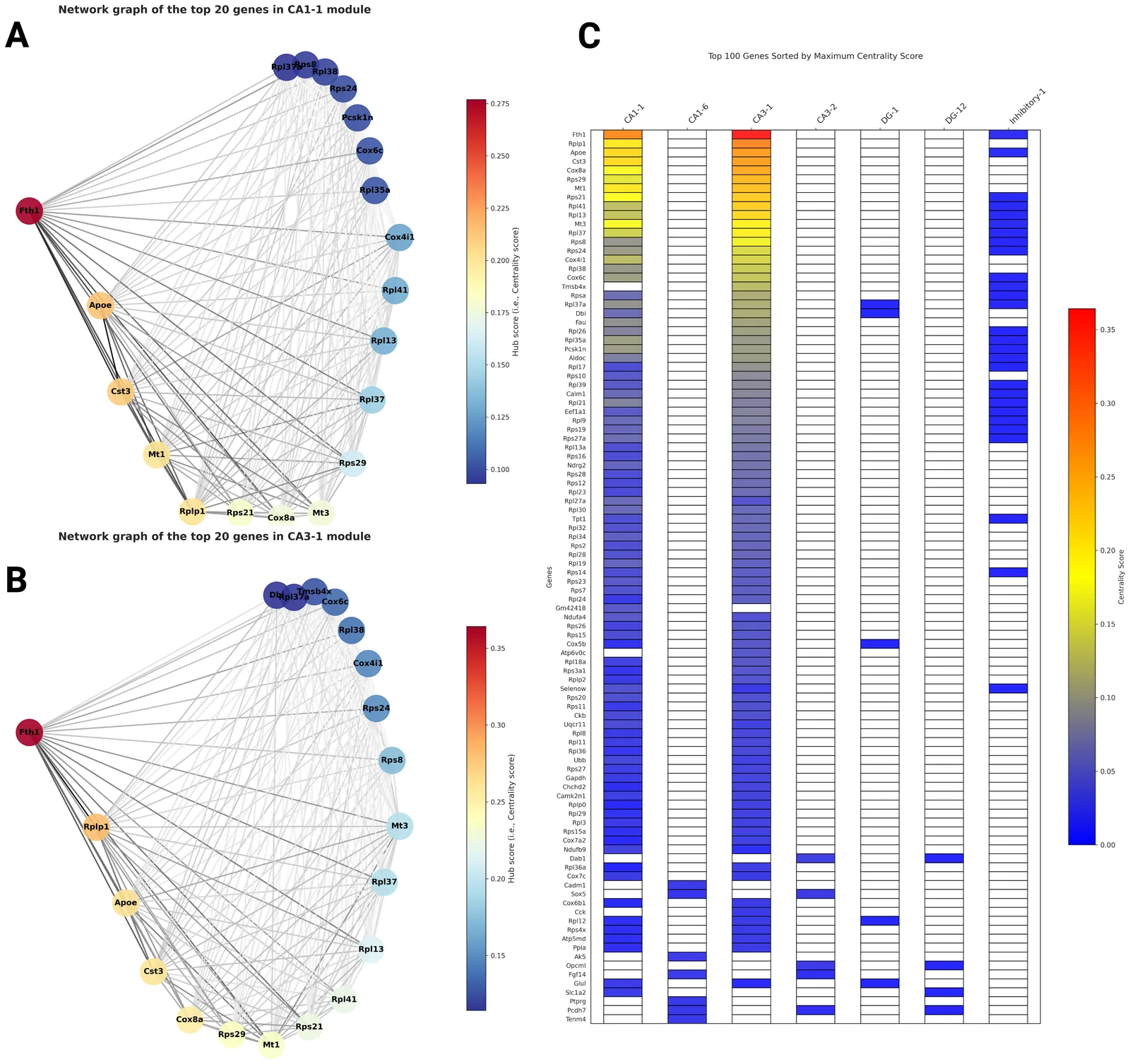
*Fth1*, *Rplp1* and *Apoe* are the most highly connected genes in the co-expression modules most affected by *Cd47* loss. Co-expression hub genes were identified using degree centrality, a metric that quantifies a gene’s connectivity within a co-expression network (mofigudule). Genes with higher centrality score (red/orange) are more connected within their module. Degree centrality measures how broadly the expression of a gene co-varies with other module members; it does not measure transcriptional regulation or protein–protein interaction. The analysis highlights central genes within specific, highly co-expressed modules and identifies hub genes shared across multiple neuronal populations. (**Panels A-B) Co-expression networks of highly co-expressed modules.** These panels visualize the co-expression networks for the top 20 hub genes in modules CA1–1 **(A)** and CA3–1 **(B)**, which were selected due to their exceptionally strong internal co-expression, i.e., hub score or equivalently centrality score (around 10x higher degree of centrality compared to other modules). Nodes represent genes, and the connecting edges are weighted by the soft-thresholded correlations of co-expression strength, with thicker (darker) edges indicating a stronger relationship. Both modules showed strong preservation when their network structure was re-evaluated using within-mouse metacells (*Z*_summary_ = 35.85 for CA1–1 and *Z*_summary_ = 25.58 for CA3–1; Table D in S1 Appendix), supporting the robustness of the network topology on which the hub-gene analysis depends. **(Panel C) Ranking of Pan-Neuronal Hub Genes.** This panel presents a shared ranking of the top 100 hub genes from the core, highly overlapping neuronal modules, including those affected by genotype such as CA1–1, DG-1, CA3–1, and Inhibitory-1. The gene ranking is based on the maximum centrality score genes across these modules. This approach highlights genes occupying highly connected positions across different neuronal cell types. *Fth1*, *Rplp1*, and *Apoe* emerged among the prominent pan-neuronal hub genes. The strong preservation of CA1–1 and CA3–1 further supports the robustness of the module topology within which these genes were identified as highly connected hubs.

Consistent with Approach 1, *Apoe* and *Rplp1* were identified in Approach 2 as hub genes within the *Cd47*-sensitive modules CA1–1 and CA3–1 (Fig 6C), extending the volcano plot findings from single-gene changes to coordinated module-level programs. The two approaches are applied to the same nuclei and the same normalised expression matrix, and are therefore not statistically independent. They do, however, measure different properties of that matrix: Approach 1 tests whether a gene’s mean expression differs between groups, whereas Approach 2 asks whether its expression co-varies with many other members of its module. Neither property implies the other—genes with large expression differences are frequently peripheral in the network, and highly central genes frequently show no difference in mean expression—so the co-occurrence of both in *Apoe* and *Rplp1* is informative, while corroborating rather than replicating the single-gene result. In the baseline genotype comparison, the positive Δ correlation of CA1–1 and CA3–1 in Fig 3B was accompanied by the upregulation of *Apoe* and *Rplp1* observed in the corresponding volcano plots in Fig 2C and Fig A in S1 Appendix, and this difference was recovered at the level of the mouse (Fig F in S1 Appendix). Furthermore, in the KA3 versus vehicle comparison, the negative Δ correlation of these same modules in Fig 3C was accompanied by the downregulation of *Apoe* and *Rplp1* observed in the corresponding volcano plots of Approach 1 (Fig D in S1 Appendix). Together, these findings indicate that the differential expression of *Apoe* and *Rplp1* is embedded in broader *Cd47*-sensitive co-expression states rather than being isolated transcriptional changes.

### Analysis of variation and distribution similarity across conditions (Approach 3)

Finally, we systematically compare estimated whole-transcriptome distributions across all genotype-treatment groups (Fig 7), to identify pairs exhibiting the highest similarity. Of the fifteen pairwise comparisons, our analysis revealed that the distributions of the *Cd47* KO-Veh and WT-KA3 groups were the most similar to each other. This finding indicates that deletion of *Cd47* under basal conditions produces a transcriptomic profile closely resembling that of wild-type cells exposed to mild excitotoxic stress induced by low-dose kainic acid. Consistent with this ranking, the mouse-level genotype contrast within the KA3 condition was null in every cell type (Fig F in S1 Appendix): once KA3 has been applied, WT and *Cd47* KO hippocampal transcriptomes are not distinguishable at the level of the animal. It is worth noting that the Wasserstein distance is a deterministic metric between fully estimated probability distributions, not a sample-level test statistic amenable to permutation-based inference. Each distribution integrates information from thousands of individual nuclei via kernel density estimation over the diffusion manifold, and the resulting pairwise distance is an exact quantity given these inputs.

**Fig 7:**
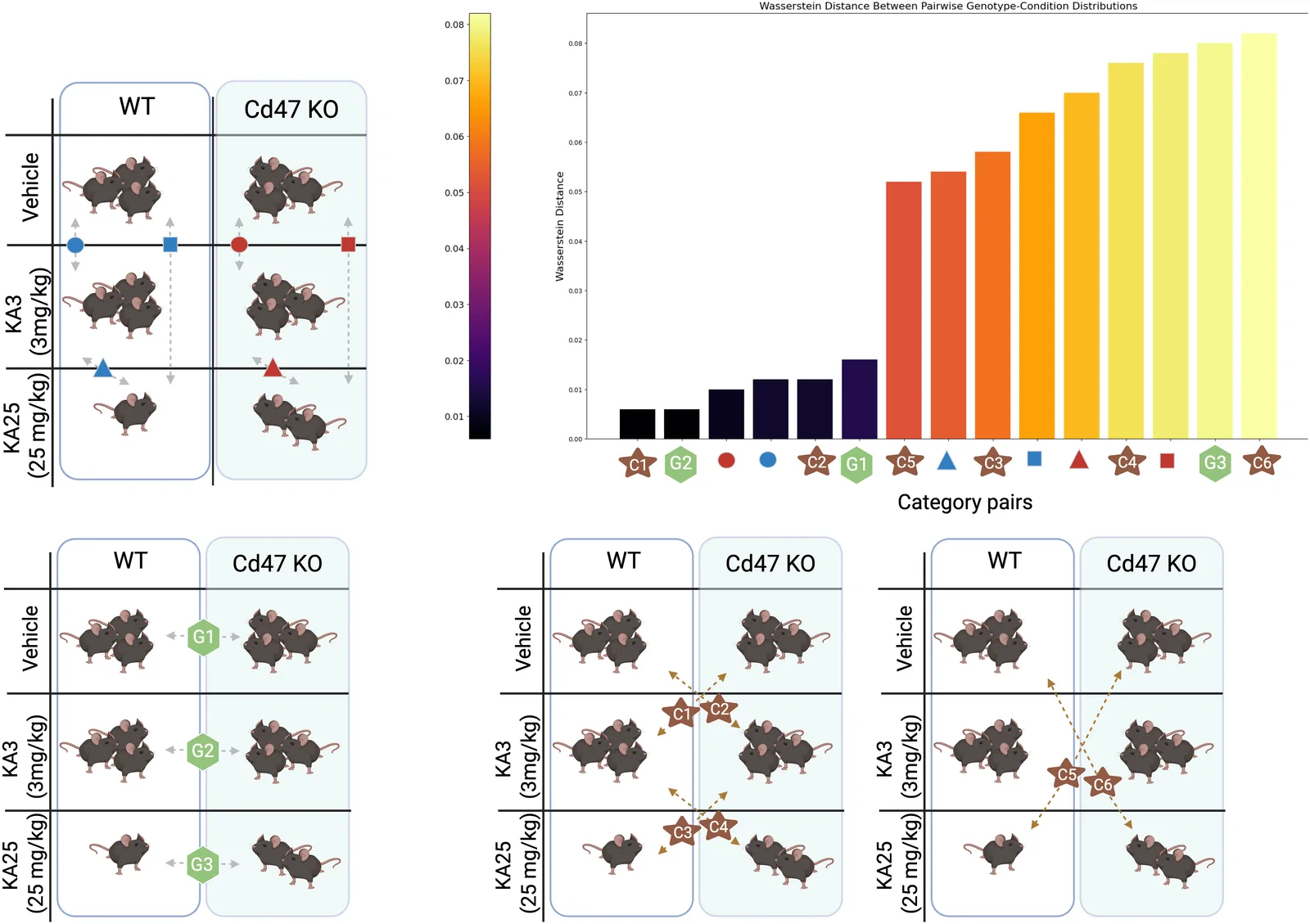
Wasserstein distance between distributions of genotype–condition pairs. Each bar represents one of the 15 unique pairwise comparisons between experimental groups and quantifies the similarity between hippocampal response distributions derived from gene transcription profiles. Distributions were estimated using kernel density estimation (KDE), and pairwise differences were computed using the normalized 1-Wasserstein distance. Distances were normalized to the [0, 1] range, with lower values indicating greater similarity and higher values indicating greater divergence between cellular response profiles. Symbols below the bars identify the comparison type shown in the schematic panels: green hexagons (G1–G3) denote Genotype contrasts within the same treatment condition, blue symbols denote treatment contrasts within WT, red symbols denote treatment contrasts within *Cd47* KO, and brown stars (C1–C6) denote Cross-genotype, cross-treatment comparisons. Created in BioRender. Kiamari, **M.** (2026) https://core.local.biorender.dev/api/short-link/6fahj39.

The strength of our finding lies in the comparative ranking across all fifteen pairwise distances, and in its consistency with Approaches 1 and 2. These approaches are complementary rather than independent: all three are applied to the same nuclei, so their agreement indicates that the conclusion that *Cd47*-deficient cells at baseline occupy a transcriptional state resembling mild excitotoxic stress is stable across three different analytical treatments of the data, not that it has been independently replicated (see Limitations and scope of inference).

## Discussion

*Cd47* is ubiquitously expressed across the majority of resident cells of the CNS where it plays a crucial physiologic role in cell-to-cell communication through the interaction with its receptor SIRPα. Pathological involvement of *Cd47* has been proposed in some neurological diseases based on data from complex and often chronic models, including neurodevelopmental disorders [8,35], autism [36], stroke [12,37], and Alzheimer’s disease [38], that confound interpretation of the contribution of neuronal, glial, and/or immune cell *Cd47*. We selected the simpler model of acute, direct excitotoxic stress to investigate cell type-specific transcriptomic differences associated with *Cd47* loss in hippocampal neurons and glia in vivo. We applied a three-tiered framework integrating single-gene, gene-module (i.e., clusters of genes), and whole-transcriptome–level analyses. By analogy with the established “don’t eat me” function of CD47, these layered computational analyses are consistent with a *Cd47*-dependent “don’t stress me” role in neuronal cells under both homeostatic and excitotoxic conditions. We offer this as an organizing hypothesis for future work rather than a demonstrated mechanism; the scope and limits of inference are set out in the following subsection.

The principal observation of this study is that *Cd47*-deficient hippocampal cells at baseline transcriptionally resemble wild-type cells under mild excitotoxic stress (as seen in Fig 7). The importance of this lies in its implication for disease: if *Cd47* loss lowers the threshold for stress-induced transcriptional change, it could be relevant to the progression of disorders driven by excitotoxicity, such as epilepsy, stroke [12,37], and neurodegeneration. Our multi-scale analytical approach allowed us to not only identify this global shift but also to uncover the specific gene sets and highly connected genes associated with this state.

The baseline state in *Cd47* KO animals is characterized by convergent transcriptional differences across cell types. Neurons and glia show higher expression of transcripts annotated to antioxidant defence and lipid metabolism, evidenced by the broad upregulation of genes like *Apoe, Cst3, Mt1, and Ptgds*, together with lower expression of synaptic and splicing-related transcripts (*Ttr, Pla2g3, Doc2b, Srsf7*) (as seen in Fig 2C and Fig A in S1 Appendix) [39–43]. Glial cells show related differences; for instance, microglia have higher expression of *Apoe, Clu, Mt1, Camk1d* and lower expression of complement-pathway transcripts (*C1qa, Selplg*) [44–47], and oligodendrocytes show differences in myelin and cytoskeletal transcripts (*Mylk, Gpm6a, Sept4*) (as seen in Fig A in S1 Appendix) [48].

Under excitotoxic challenge the genotype contrast changes character. Following low-dose KA3, *Cd47* KO neurons show higher expression of genes involved in AMPA receptor trafficking (*Prrt1*) and cell signal suppression (*Sag*), alongside genes implicated in synaptic plasticity and extracellular matrix modulation (*Chpf2, Dynlt1a, Safb*) (as seen in Fig 2D and Fig B in S1 Appendix). Under severe excitotoxicity (KA25), the descriptive picture is dominated by genes like *Ttr, Mt1, Fkbp5*, and *Apoe*, coupled with downregulation of essential cytoskeletal and synaptic components (*Actb, Camk1d*) (Fig 2E and Fig C in S1 Appendix) [49–51]. Notably, the glial inflammatory signature in *Cd47* KO mice is comparatively muted, consistent with *Cd47*’s known role in modulating macrophage and microglia engagement [9,52,53], and, because tissue was collected 12 hours after injection, later time points would be needed to determine whether this reflects a genuine attenuation or simply a delayed secondary immune reaction.

To understand how these transcriptional differences are organized, we turned to network analysis (Approach 2). WGCNA revealed modules linking translational control, bioenergetics, and synaptic regulation, whose co-expression structure differs by genotype (Figs K–M in S1 Appendix). This approach allowed us to move beyond individual gene changes to identify the most highly connected genes of the hippocampal excitotoxic response. Analysis of these modules identified a distinct set of co-expression hub genes—most notably *Fth1*, *Rplp1*, and *Apoe*—whose annotated functions suggest the core biological axes relevant to this stress (Fig 6). We discuss each below as a hypothesis about why a gene with that annotated function might occupy a central network position.

*Fth1*: as a central hub gene, *Fth1*’s annotated role is to coordinate the cell’s defense against iron-induced oxidative stress [54,55], a major consequence of Kainic Acid (KA) toxicity. KA-induced excitotoxicity leads to a massive increase in reactive oxygen species [56], and free iron acts as a powerful catalyst in these damaging reactions [57]. *Fth1* is a key effector of iron homeostasis that limits iron-catalyzed oxidative damage by sequestering iron, and its expression is regulated in response to iron and stress cues [54,55]. Moreover, ferritin turnover through NCOA4-dependent ferritinophagy can regulate intracellular iron bioavailability, which is mechanistically linked to susceptibility to iron-dependent oxidative injury/ferroptosis [58,59]. In *Cd47* knockout contexts, CD47 signaling is known to modulate cellular stress-response pathways (including NO/cGMP signaling and stress-linked programs such as autophagy), which could alter baseline homeostasis and the capacity to adapt to additional insults [47]. Therefore, based on *Fth1*’s high centrality in the hippocampal network, we hypothesize that *Cd47* deletion may shift the set-point of iron-handling responses (e.g., ferritin expression/turnover), either priming protection or increasing vulnerability to KA-driven oxidative stress and ferroptosis-like injury [58,59]. This is directly testable by measuring labile iron, ferritin protein and lipid peroxidation in *Cd47* KO hippocampus after KA.

*Rplp1*: the high centrality of *Rplp1*, a core component of the ribosome, leads us to hypothesize that the regulation of protein synthesis itself [60] is a relevant control point for surviving excitotoxic stress. In the face of a severe insult like KA, a cell must rapidly shift its resources, shutting down the production of non-essential proteins while prioritizing the synthesis of critical survival factors [61–63] like heat shock proteins and antioxidant enzymes [64–66]. *Rplp1*’s position as a co-expression hub places it among the transcripts whose abundance co-varies most tightly with the rest of the module [67,68]. Based on this network position, we hypothesize that the *Rplp1*-centred module may reflect coordinated regulation associated with the translational response and may contribute to cellular resilience [63,69], and that altered regulation of this module in *Cd47* knockout cells may contribute to differences in susceptibility to KA. This hypothesis can be tested directly by assessing ribosome occupancy or nascent protein synthesis in *Cd47* KO hippocampus following KA exposure.

*Apoe*: *Apoe*’s role as a top-ranked hub gene is consistent with the importance of lipid logistics, structural repair, and neuron-glia communication in the response to KA-induced injury [70,71]. Primarily produced by astrocytes, *Apoe* is responsible for transporting cholesterol and lipids needed to repair the widespread membrane damage caused by excitotoxicity [72]. Its high centrality signifies that its expression co-varies with many module members spanning lipid delivery, clearance of cellular debris, and modulation of the neuroinflammatory response [45,73]. Whether *Apoe* coordinates this program or instead tracks it is a hypothesis generated by the network analysis rather than a conclusion drawn from it, and it is experimentally tractable. In the *Cd47* knockout genotype, where the “don’t eat me” signal to microglia is absent, the dynamics of debris clearance after cell death are plausibly altered, which makes *Apoe*-associated repair and cleanup processes a priority for direct test: lipidomic profiling, phagocytic-flux assays and cytokine measurement in *Cd47* KO hippocampus after KA would each discriminate a coordinating role from a correlative one. We identify this as the most informative next step arising from the present dataset.

Among these hub genes, *APOE* is the strongest Genome-Wide Association Study (GWAS) risk factor for late-onset Alzheimer’s disease [74,75], providing direct convergence between our network analysis and human disease genetics. Neither *FTH1* nor *RPLP1* is an established GWAS locus, consistent with their roles as effector and housekeeping genes under evolutionary constraint rather than common variant-driven risk factors. This complementarity underscores that co-expression hub analysis captures genes central to acute transcriptional stress responses that may not be detected by genetic association approaches.

### Limitations and scope of inference

This study is a transcriptomic survey and its conclusions are correspondingly bounded.

**Sample size.** Group sizes are three animals per genotype for Veh and KA3, and one WT and two KO for KA25. With one WT and two KO animals the smallest attainable permutation p-value is 0.333, so the KA25 genotype contrast cannot support inferential claims and is presented descriptively throughout. Single-nucleus sequencing yields thousands of transcriptomes per mouse and WGCNA constructs networks across all 15 mice simultaneously, so KA25-associated patterns are constrained by the full experimental design.

**Unit of replication.** Nuclei from the same animal are not independent. All single-gene contrasts reported as significant are tested at the level of the mouse (Fig F in S1 Appendix), and module eigengenes are analysed with mouse-level linear models (see Methods). Co-expression networks were constructed at the level of the nucleus, because a within-cell-type correlation network estimated from 15 samples would be underdetermined; module–trait correlation p-values computed at the nucleus level are consequently anti-conservative and are not used for inference. Module stability was instead assessed by re-evaluating the fixed module assignments in a metacell representation of the same data (Table D in S1 Appendix).

**Nuclei recovery.** Nuclei recovery differed across animals, ranging from 571 to 7,295 nuclei per mouse, and was most uneven in the vehicle condition, where the three WT animals yielded 571, 816, and 7,295 nuclei (Table A2 in S1 Appendix). Per-nucleus transcript capture was far more uniform across animals (median 3,566–5,536 UMIs; 1,792–2,508 genes), suggesting that the dominant source of variation was the number of nuclei recovered rather than transcript capture within each nucleus. Because pseudobulk counts scale with the number of contributing nuclei, all mouse-level expression testing used library-size normalisation. In addition, within each treatment condition, KO and WT nuclei were downsampled to the smaller group before network construction so that the co-expression analyses were not driven by genotype-associated differences in nucleus number. Median UMIs per nucleus also differed systematically among cell types (CA3 ≈ 10,500; CA1 ≈ 7,400; inhibitory neurons ≈ 6,200; DG ≈ 5,200; astrocytes ≈ 2,100; oligodendrocytes ≈ 1,800; microglia ≈ 1,400), consistent with expected biological and technical differences in transcript capture across cell types. Per-sample sequencing metrics, including total reads and mean reads per nucleus, are reported in Table A1 in S1 Appendix, and within-cell-type coverage is reported as UMIs and detected genes per nucleus in Table A4 in S1 Appendix. Sequencing depth was therefore sufficient across all cell types, and nucleus number was the principal quantity governing the resolution of the co-expression analysis: only three of the 105 mouse × cell-type strata fell below 50 nuclei, all in the single low-recovery vehicle WT animal (*WT_Veh_3*: CA3 *n* = 45, inhibitory *n* = 48, microglia *n* = 27). Microglia were the least abundant population overall (3,086 nuclei in total; 198 in the KA25 KO group and 335 in the vehicle WT group), and microglial modules were therefore interpreted with lower confidence than neuronal modules; the module preservation analysis in Table D in S1 Appendix provided the basis for this distinction.

**Non-independence of the three analytical approaches.** Approaches 1–3 were applied to the same underlying single-nucleus transcriptomic dataset and therefore do not constitute independent biological evidence. They do, however, interrogate distinct mathematical properties of that dataset: differential expression evaluates per-gene shifts in mean abundance, the co-expression analysis evaluates second-order correlation structure among genes, and the distributional comparison evaluates the shape of the whole-transcriptome distribution. These properties are not redundant, in that a gene may shift in mean abundance without altering its module correlations, and a module may reorganise its correlation structure without any constituent gene reaching significance. Agreement across the three therefore indicates that a result is not an artefact of a single analytical representation; it does not provide biological replication, which would require an independent cohort.

**Absence of functional validation.** No protein-level, morphological, or electrophysiological measurement was made in the sequenced animals. Statements about resilience, vulnerability, activation, remodeling and stress sensing are hypotheses generated by the transcriptomic data, not demonstrated mechanisms.

**Dose design.** Two doses cannot resolve the shape of the dose–response relationship. Whether *Cd47* loss shifts the excitotoxic dose–response curve leftward, as the baseline transcriptomic state suggests, requires a graded dose series.

**Cell types analysed.** Seven populations with sufficient nuclei for within-cell-type network construction were analysed. Clusters that did not meet this criterion were not carried forward.

**Sex.** Both sexes were included and animals were age-matched across genotype and treatment, but with one to three animals per group exact sex matching within every group is not attainable and sex cannot be tested formally as a biological variable. To limit the risk that sex imbalance generates spurious genotype differences, sex-linked genes were excluded from the co-expression network input, and we note that none of the three genes on which the central conclusions rest (*Fth1*, *Rplp1*, *Apoe*) is sex-linked.

## Materials and methods

### Ethics statement

All experimental mouse protocols performed at Stanford University were approved by the Stanford University Administrative Panel on Laboratory Animal Care (APLAC; protocol #31890) and were carried out in accordance with the National Institutes of Health Guide for the Care and Use of Laboratory Animals. All efforts were made to minimize animal suffering and to reduce the number of animals used. Mice reaching predefined humane endpoints (status epilepticus), or at study termination, were euthanized by isoflurane inhalation overdose followed by a secondary physical method to ensure death, in accordance with APLAC-approved protocols.

### Animals

All mice were maintained on a 12/12 light/dark cycle at room temperature (20–23 °C) and relative humidity of 50%. Food and water were provided ad libitum. C57BL/6 mice and *Cd47* KO (B6.129S7-*Cd47*^tm1Fpl^/J) were purchased from Jackson Laboratory. Adult mice of both sexes, approximately 3 months of age and weighing 20–25 g at the time of injection, were used. Age and weight were matched across genotype and treatment groups. The cohort comprised 8 males and 7 females: 4 males and 3 females among the 7 WT animals, and 4 males and 4 females among the 8 *Cd47* KO animals. The composition of each genotype–treatment group was as follows: Veh, WT 2 male/1 female and KO 1 male/2 female; KA3, WT 2 male/1 female and KO 2 male/1 female; KA25, WT 1 female and KO 1 male/1 female. The sex of every individual animal is reported in Table A1 in S1 Appendix. Sex is therefore approximately balanced at the level of genotype, but with group sizes of one to three animals it could not be matched exactly within every genotype–treatment group, and the design does not permit formal testing of sex as a biological variable or the inclusion of sex as a covariate. Two consequences are stated explicitly. First, the vehicle arm, which carries the central baseline comparison, has reciprocal sex ratios in the two genotypes (2 male/1 female in WT versus 1 male/2 female in KO). Second, the single WT animal in the KA25 arm is female, so the KA25 genotype contrast is confounded with sex; this contrast is reported descriptively only throughout, for this reason as well as on grounds of sample size. To limit the risk that sex imbalance generates spurious genotype differences, sex-linked genes were excluded from the expression matrix used for co-expression network construction, and we confirmed that none of the genes on which the conclusions of this study rest, including *Fth1*, *Rplp1* and *Apoe*, is sex-linked.

### KA treatment

Kainic Acid (KA), a potent glutamate receptor agonist that serves as a well-established tool for inducing seizures and hippocampal excitotoxicity [76] was purchased from Millipore Sigma (Catalog 420318) and dissolved in 0.9% of saline solution. The KA model effectively mimics the progression from neuronal hyperexcitability to cell death, likely including stress-response mechanisms of the hippocampus [77]. Animals received a single intraperitoneal (IP) injection of KA3 (3 mg/kg), KA25 (25 mg/kg), or vehicle (0.9% saline solution). Seizure activity induced by both KA doses was independently validated (Fig O in S1 Appendix).

**Rationale for dose selection.** Susceptibility to kainic acid is strongly strain-dependent, and C57BL/6 mice are relatively resistant to KA-induced excitotoxic neuronal death: 129/SvEMS and FVB/N mice show CA1 and CA3 excitotoxic cell death after systemic KA, whereas C57BL/6 and BALB/c mice exhibit comparable seizures but excitotoxic cell death only at very high doses and in restricted regions [78]. Published seizure responses to systemic KA in C57BL/6J mice also vary. Ferraro and colleagues reported that C57BL/6J mice remained seizure-free at 25 mg/kg administered subcutaneously and that only 25% exhibited tonic–clonic seizures at 30 mg/kg, whereas DBA/2J mice seized at both doses [79]. In contrast, Tse and colleagues used a single 20 mg/kg i.p. dose to induce status epilepticus in C57BL/6J mice, with approximately 21% mortality [80]. Adult C57BL/6J mice have also been administered 35 mg/kg i.p. for neuropathological studies, whereas the dose was reduced to 20 mg/kg in aged animals to limit excessive mortality [81]. Because of this variability, we did not rely on a single published seizure threshold. We selected 25 mg/kg as a dose within the range used to induce seizure activity and neuropathological effects in C57BL/6J mice and verified its convulsive effect behaviourally in our own colony. We selected 3 mg/kg as a lower, subconvulsive exposure—approximately one-eighth of the KA25 dose and below the 5 mg/kg increments used in repeated-low-dose status epilepticus protocols [80]—intended to provide a lower excitotoxic challenge without overt seizure activity. The behavioural distinction between these doses was evaluated in an independent cohort (Fig O in S1 Appendix): modified Racine scores differed significantly among Veh, KA3 and KA25 over the first hour (Kruskal–Wallis *H* = 9.415, df = 2, *p* = 0.009), with the highest scores observed after KA25. KA25 was also associated with reduced path length and locomotor speed and increased freezing episodes, whereas KA3 did not differ detectably from vehicle on the behavioural measures examined. Neither dose was intended to define a maximum-tolerated or median-lethal dose, and the present experiment was not designed to establish a full dose–response relationship.

### Single-nucleus RNA sequencing

#### Data collection and pre-processing.

7 wild-type (WT) and 8 *Cd47* knockout (KO) mice were treated with vehicle (Veh; n = 3 WT and n = 3 KO), low-dose kainic acid (KA3; 3 mg/kg; n = 3 WT and n = 3 KO), or high-dose kainic acid (KA25; 25 mg/kg; n = 1 WT and n = 2 KO)—(Fig 2A). These 15 animals are exactly the animals for which raw sequencing reads and processed count matrices are deposited in GEO, and every analysis reported in this manuscript was run on that deposited dataset (see Data availability). Mice were sacrificed 12 hours after IP injection. Hippocampal tissue was rapidly collected and processed for nuclei isolation [82]. After isolation, nuclei were captured and sequenced by the Stanford Functional Genomics Facility. Capture was performed with 10X Genomics Controller device (Pleasanton, CA, United States) following manufacturer’s recommendations while library preparation was performed according to the 10x Genomics Chromium Single Cell 3’ v3 Reagent Kit and sequenced on HiSeq 4000 instrument (Illumina, San Diego, CA, United States). Reads were subsequently processed using the 10X Genomics Cell Ranger 7.0.1 analytical pipeline with default settings and aligned to the mm10 genome. Following data collection, we removed empty droplets, identified and removed low-quality nuclei based on standard quality-control metrics (mitochondrial gene fraction and number of detected genes) using Scanpy [83], and detected and excluded computational doublets using Scrublet [84].

Sequencing and alignment metrics for each of the 15 libraries, generated by Cell Ranger, are reported in Table A1 in S1 Appendix: libraries yielded 30.8–586.8 million reads (22,489–42,623 mean reads per called cell), 32–47% sequencing saturation, 50–59% of reads mapped confidently to the transcriptome, and 40–58% of reads in cells. The fraction of reads in cells is lower than the value typical of whole-cell preparations, as expected for single-nucleus libraries in which ambient RNA contributes appreciably to the droplet background. For this reason we did not use Cell Ranger cell calling. We instead began from the raw feature–barcode matrix and applied our own filters: per nucleus we computed the number of detected genes, the total UMI count and the percentage of mitochondrial counts in Scanpy, retaining nuclei with more than 150 and fewer than 7,000 detected genes and fewer than 5% mitochondrial counts, and examining quality-control distributions before and after filtering. Doublets were identified independently within each sample with Scrublet. Because our filters were applied to the raw matrix rather than to the Cell Ranger cell calls, the Cell Ranger “estimated number of cells” is a parallel estimate rather than a stage in this pipeline, and per-mouse counts are not nested within it. After quality control, clustering and cell-type annotation, 64,569 nuclei were assigned to the seven cell populations analysed. One consequence of filtering from the raw matrix is worth making explicit. Library *Veh_ko2* (mouse KO_Veh_1) was over-called by Cell Ranger at 26,091 cells with the lowest mean reads per called cell (22,489) and correspondingly low Cell Ranger medians of 1,131 genes and 2,150 UMIs per cell. After removal of empty and low-complexity droplets, the 7,097 retained nuclei from this mouse have median values of 2,508 genes and 5,536 UMIs, the highest of any animal in the cohort. The apparent discrepancy between the Cell Ranger and post-filtering medians for this library therefore reflects the removal of background droplets, not a difference in sequencing depth. Per-mouse nuclei counts and depth are given in Table A2 in S1 Appendix, nuclei counts for every mouse and cell type in Table A3 in S1 Appendix, within-cell-type depth in Table A4 in S1 Appendix, and nuclei and depth per cell type, genotype and treatment group in Table A5 in S1 Appendix. A mapping between Cell Ranger library identifiers and the mouse identifiers used throughout is given in Table A1 in S1 Appendix.

Subsequently, we normalized counts to 10,000 UMIs per nucleus, log1p-transformed them, and applied principal component analysis (PCA) for dimensionality reduction and used the Leiden algorithm to identify cell clusters based on transcriptional similarity. Clustering was unbiased: no cell population was specified in advance, and the seven populations reported emerged from this procedure. To assign biological identities to these clusters, we first performed differential expression analysis between clusters to identify unique marker genes for each group, using a Wilcoxon rank-sum test of each cluster against all others (Scanpy rank_genes_groups). By comparing these marker genes to the Allen Institute’s mouse brain atlas (Whole Cortex & Hippocampus 10x – Transcriptomics explorer) [17] and to canonical or region-enriched markers (*Slc17a7* as a pan-excitatory marker, with subregional identity supported by *Fibcd1* for CA1 and *Cabp7*/*Bok* for CA3; *Prox1* and *Dock10* for DG; *Gad1*/*Gad2* for inhibitory neurons; *Aqp4*/*Slc1a2* for astrocytes; *Plp1*/*Mbp* for oligodendrocytes; *Cx3cr1*/*P2ry12* for microglia), we annotated major neuronal populations (CA1, CA3, DG, and inhibitory neurons) and glial cell types (astrocytes, oligodendrocytes, and microglia), as visualized in the UMAP in Fig 2B. The seven populations carried forward are those that could be confidently assigned an identity and that contained enough nuclei in every genotype × treatment group to support within-cell-type co-expression network construction. Clusters that failed either criterion were not analysed further. Because excitotoxic stress is likely to affect these populations as well, we note in Limitations that their responses are not captured by this study. Nuclei counts for every mouse and every analysed cell type are given in Tables A1-A5 in S1 Appendix.

### Approach 1

#### Nucleus-level differential expression analysis.

To identify gene-level perturbations attributable to *Cd47* loss under each treatment context, we conducted differential expression analyses comparing WT and KO within the vehicle, subconvulsive KA3 (3 mg/kg), and convulsive KA25 (25 mg/kg) conditions. Statistical testing with multiple hypothesis correction yielded robust sets of up- and down-regulated genes, which were summarized by effect size and statistical significance to highlight the most strongly perturbed transcripts. These contrasts nominate candidate genes whose expression is selectively sensitive to genotype across increasing neuronal stress levels and form a gene-centric foundation for mechanistic interpretation (Fig 1B). Volcano plots were generated via differential expression analysis to identify the statistically significant up-regulated and down-regulated genes (**Approach 1**, Fig 2C-2E, Figs A-E in S1 Appendix). Nuclei from the same animal are not independent biological replicates. We therefore state explicitly which unit of replication underlies each analysis. The volcano plots in Fig 2C–2E and Figs A-E in S1 Appendix are nucleus-level, computed with the Wilcoxon rank-sum test in Scanpy (rank_genes_groups) [83] on log1p CP10K values with Benjamini–Hochberg correction, treating each nucleus as an observation, and are presented as descriptive summaries of the cellular data and as the basis for gene nomination.

#### Mouse-level pseudobulk differential expression analysis.

For each mouse and each annotated cell type, raw counts were summed across all nuclei assigned to that cell type, yielding one pseudobulk profile per mouse per cell type. Genes with fewer than 10 counts in at least three animals were removed. Because the number of nuclei contributing to each profile differs substantially between animals, library size was normalised before testing; this correction is essential rather than cosmetic, since raw pseudobulk sums scale with the number of contributing nuclei. *Cd47* KO was compared with WT within each treatment condition separately, and *p*-values were adjusted for multiple testing across genes within each cell type using the Benjamini–Hochberg procedure. Results for all seven cell types and all three conditions are shown in Fig F in S1 Appendix. We note one structural limitation of the design. In the high-dose group (*n* = 1 WT and *n* = 2 KO) the exact permutation distribution contains only 3 arrangements, so the smallest attainable *p*-value is 1/3≈0.333, before any multiple-testing correction. No result at this dose can reach conventional significance regardless of effect size, and the KA25 genotype contrast is therefore reported descriptively only.

### Approach 2

#### Weighted gene co-expression network analysis.

Weighted Gene Co-expression Network Analysis (WGCNA) [31] is a robust method designed to detect groups of genes (modules) that exhibit coordinated expression patterns across samples. In brief, WGCNA involves constructing a weighted correlation network based on pairwise gene expression similarities, identifying modules of highly interconnected genes, summarizing these modules through representative eigengenes, and associating these modules with external biological traits or cell-type annotations. All analyses were performed using the pyWGCNA implementation of WGCNA in Python [32], unless otherwise specified. Networks were constructed on nucleus-level, library-size-normalised, log1p-transformed expression within each cell type, with one row per nucleus; we note that nuclei from the same animal are not independent, so the nucleus-level network is used for module *detection*, an unsupervised estimation of gene–gene correlation structure in which no genotype or treatment label is used and for which the number of observations is an advantage. Within each treatment condition, KO and WT nuclei were randomly downsampled to the smaller group before network construction, so that every network is genotype-balanced at every dose. Initially, we constructed a gene co-expression network by computing pairwise correlations among gene expression profiles across samples. To enhance robustness against outliers and deviations from normality, we utilized the biweight mid-correlation as our similarity measure. The resulting similarity matrix was then transformed into a weighted adjacency matrix by applying a soft thresholding power, selected to approximate a scale-free network topology. Subsequently, the adjacency matrix was converted into a topological overlap matrix (TOM), which captures both direct and indirect relationships between genes, providing a comprehensive measure of network connectivity. We then performed hierarchical clustering using the TOM-based dissimilarity measure (1–TOM) and identified gene modules through the dynamic tree-cutting algorithm. To summarize the expression patterns within each module, we calculated module eigengenes, defined as the first principal component of the gene expression data within each module. The input matrix, gene filtering and network parameters were as follows. Gene filtering used the full post-quality-control gene set for each cell type; the PyWGCNA preprocess() step removed genes and observations exceeding its default missing-value tolerance, and the retained set for each cell type was also used as the background (universe) for enrichment testing. The number of nuclei used for network construction after genotype balancing, the number of genes retained, and the number of modules identified for each cell type are summarized in Table 1. The soft-thresholding power was selected automatically for each cell type using the scale-free topology criterion implemented in PyWGCNA, searching β=1–19. The selected power was β=2 for CA1, CA3, inhibitory neurons, and oligodendrocytes (scale-free fit *R*^2^ = 0.98–0.99) and β=4 for dentate gyrus (*R*^2^ = 0.91), with the same automated criterion applied to astrocytes and microglia. Modules were detected by dynamic tree cutting on 1−TOM and subsequently merged using mergeCloseModules at an eigengene dissimilarity threshold of 0.2, such that modules whose eigengenes correlated at *r* > 0.8 were combined. This procedure yielded 110 modules across the seven cell types.

**Table 1:** Summary of cell-type-specific co-expression network construction. The table reports the number of nuclei used after genotype balancing within treatment conditions, the number of genes retained for network analysis, and the number of co-expression modules identified for each cell type.

Cell type	Nuclei (balanced)	Genes retained	Modules
CA1	9,468	22,010	15
CA3	4,964	19,068	39
DG	14,600	24,106	2
Inhibitory	6,018	22,327	12
Astrocytes	3,776	21,520	15
Oligodendrocytes	9,920	23,127	7
Microglia	2,422	20,735	20
**Total**	**51,168**	—	**110**

Table B in S1 Appendix lists the number of genes assigned to each of the genotype- and KA-associated modules carried forward for downstream analysis. Gene membership for every module is available in the code repository (see Data availability). Co-expression network analyses were performed using PyWGCNA [32]. Network stability was evaluated using the module preservation analysis described below.

#### Module preservation analysis.

Because the co-expression networks were constructed from individual nuclei, we assessed whether the resulting modules retained their co-expression structure in a less sparse representation of the same data. The original module assignments were held fixed during this sensitivity analysis; modules were neither redefined nor renumbered.

**Metacell construction.** Metacells were generated separately within each mouse × cell-type combination, ensuring that nuclei from different animals were never aggregated into the same metacell and that mouse identity was retained. Within each combination, nuclei were partitioned by *k*-means clustering using the first 30 principal components of the PCA embedding into approximately *n*/15 clusters, where *n* denotes the number of nuclei in that mouse × cell-type combination, targeting approximately 15 nuclei per metacell. Raw counts were summed within each partition, followed by library-size normalization and log transformation. Mouse × cell-type combinations containing fewer than 10 nuclei were excluded. Partitions were non-overlapping, such that each nucleus contributed to exactly one metacell. This procedure yielded 4,303 metacells from 64,569 nuclei and substantially reduced expression-matrix sparsity. This analysis represents an adaptation of the metacell strategy developed to obtain more robust expression profiles from sparse single-cell data and subsequently applied to single-cell co-expression network analysis [85,86].

**Preservation statistics.** Module preservation was evaluated using a permutation-based framework motivated by the network-preservation principles described by Langfelder et al. [87], with the nucleus-level expression matrix serving as the reference representation and the metacell expression matrix of the corresponding cell type serving as the test representation. For each fixed module, we evaluated four measures of module density in the metacell representation: mean absolute gene–gene correlation, mean adjacency, proportion of variance explained by the first principal component, and mean absolute eigengene-based connectivity (|*kME*|). We additionally evaluated four measures of connectivity preservation between nucleus and metacell representations: correlations of intramodular connectivity (*kIM*), |*kME*|, gene–gene correlation vectors, and adjacency vectors. Adjacencies were calculated as $|r{|}^{\beta }$, using the soft-thresholding power β specified for the corresponding cell-type network.

For each statistic *s*, the observed value was standardized relative to a null distribution generated from 50 randomly sampled gene sets of matched size,


$$\begin{array}{c} Z_{s}=\frac{\theta_{s}^{obs}-\mu_{s}^{null}}{\sigma_{s}^{null}}. \end{array}$$

A composite preservation score was then calculated as


$$\begin{array}{c} Z_{summary}=\frac{1}{2}\left(Z_{density}+Z_{connectivity}\right), \end{array}$$

where *Z*_density_ and *Z*_connectivity_ denote the medians of the four corresponding standardized statistics. Medians were used to reduce sensitivity to individual degenerate or extreme component statistics. Modules containing fewer than 10 genes were not evaluated, and those containing more than 400 genes were evaluated using a random subset of 400 module genes for computational tractability. The reference nucleus-level expression matrix was additionally limited to a maximum of 3,000 randomly sampled nuclei per cell type for preservation calculations. The resulting composite score was interpreted comparatively across modules, with positive *Z*_summary_ values indicating preservation statistics above their permutation-derived null expectations and larger positive values providing stronger evidence of preservation. Of the 110 modules, 85 (77%) had *Z*_summary_ > 2 and 49 (45%) had *Z*_summary_ > 10. The modules underpinning the principal conclusions of this study were among the highest-scoring modules within their respective cell types. A high *Z*_summary_ therefore indicates overall preservation of module coherence and connectivity structure, including similarity in intramodular connectivity, eigengene-based connectivity, gene–gene correlations, and adjacency patterns between the nucleus-level and metacell representations. Full results for all tested modules are provided in Table D in S1 Appendix. Analyses were implemented in Python using NumPy, SciPy, and scikit-learn.

#### Module-trait relationship heatmaps for different cell types.

By using using the pyWGCNA implementation of WGCNA [32] and following the identification of gene modules and the calculation of their representative eigengenes, the next critical step is to relate these modules to external biological information. This is achieved through a module-trait correlation analysis. In this context, a trait refers to any genotype-treatment pair, considering the two genotypes (WT and *Cd47* KO) and the three treatments: vehicle, KA3 (3 mg/kg), and KA25 (25 mg/kg). For each module, its eigengene—which summarizes the collective expression pattern of all genes within it—is correlated with each trait. The resulting output is a matrix of correlation coefficients and corresponding adjusted p-values, which is typically visualized as a heatmap for intuitive interpretation (see Figs G and H in S1 Appendix). The module-trait relationship heatmap serves as a powerful data reduction and screening tool, enabling the rapid identification of modules that are significantly associated with the traits of interest. Each cell in the heatmap represents the strength and direction of the association, with strong positive correlations indicating that a module’s gene expression increases with the trait, and strong negative correlations indicating the opposite. This analysis effectively transforms the large-scale, abstract network structure into a set of testable biological hypotheses, guiding downstream analyses such as functional enrichment to uncover the pathways represented by the module and the identification of central hub genes within it.

#### Mouse-level analysis of module eigengenes.

Module eigengenes were computed from the within-mouse metacell expression matrix and averaged within each animal to give one value per module per mouse. The analysis was restricted to the vehicle and KA3 conditions (*n* = 12 mice), because the KA25 arm contains one WT and two KO animals and cannot support inference, and to the 85 modules identified as preserved in the module preservation analysis. Each eigengene was fitted with ME genotype + treatment + genotype:treatment, with genotype (WT, KO) and treatment (Veh, KA3) as two-level factors, giving 8 residual degrees of freedom. *p*-values were adjusted using the Benjamini–Hochberg procedure across modules within each cell type separately for each model term. Effect sizes are reported with 95% confidence intervals. Results are reported in the Results.

#### Functional enrichment and pathway analysis.

To characterize the biological processes represented by the highly overlapping modules uncovered through our cross-module comparison, we performed an in-depth functional enrichment analysis (Fig 5 and Figs K–M in S1 Appendix). This analysis leveraged multiple databases, including Gene Ontology (GO), KEGG, WikiPathways, REACTOME, and SynGO, to map module genes to nominate biological pathways and processes. The background gene set (universe) was defined as all genes detected and retained after preprocessing and quality control in the corresponding cell type. Only modules containing ≥10 genes were considered for enrichment analysis. For each module, enrichment was assessed using the hypergeometric test with FDR correction. All analyses were performed in Python 3.11 using the gseapy package [88].

#### Co-expression hub analysis.

Co-expression hubs within each module were identified based on network centrality score [89,90] using the weighted adjacency matrix from WGCNA. Degree centrality (threshold > 0.02) was computed to suppress weak co-expression edges and highlight the most connected genes. For each module, genes were ranked by their centrality scores, and the top 20 nodes were visualized as subnetworks with edge weights proportional to co-expression strength. To identify cross-module genes, maximum centrality values were compared across neuronal modules, and the top 100 genes were selected to define pan-neuronal hub candidates (Fig 6). All network analyses were implemented in Python 3.11 using NetworkX. As noted in the Results, degree centrality in a co-expression network measures how broadly the expression of a gene co-varies with module members; it is not a measure of regulatory influence.

### Approach 3

#### Analysis of distribution similarity across conditions.

**Distribution estimations.** Burkhardt et al. [91] presented a method for estimating the distribution density of cell states derived from single-cell RNA-seq data. Instead of partitioning cells into discrete clusters, the method models the high-dimensional transcriptomic space as a smooth manifold, where biologically meaningful cell states reside. By estimating the density of cells from different experimental conditions over this manifold, the approach quantifies how the likelihood of observing specific cell states changes as a result of perturbations. The foundation of this approach is an extension of the classical kernel density estimation (KDE) method. In Euclidean space, KDE is defined as:


$$\begin{array}{c} f(x,t)=\frac{1}{N}\sum_{i=1}^{N}K(x,X_{i},t), \end{array}$$

where *x* represents a point in space, $X_{i}$‘s are the observed data points, *N* is the total number of observations, and $K(x,X_{i},t)$ is a kernel function, typically chosen to be Gaussian:


$$\begin{array}{c} K(x,y,t)=\frac{1}{(4\pi t{)}^{d/2}}\exp\left(-\frac{\|x-y{\|}^{2}}{4t}\right). \end{array}$$

To generalize kernel density estimation to a non-Euclidean space, we leverage diffusion geometry defined on a cell–cell similarity graph. This formulation is rooted in diffusion maps, which model high-dimensional data as a smooth manifold using a diffusion operator derived from the graph Laplacian [92]. Diffusion-based embeddings have been widely adopted in single-cell transcriptomics to preserve intrinsic biological structure and continuous cellular trajectories, including in PHATE, which explicitly models manifold geometry in scRNA-seq data [93]. Building on this framework, condition-specific density estimation over the diffusion manifold enables quantitative comparison of cellular state distributions across experimental perturbations [91]. The corresponding density estimate on the manifold is expressed as:


$$\begin{array}{c} f(x,t)=e^{-tL}x=\Psi \;h(\Lambda )\;\Psi^{-1}x \end{array}$$

where *L* is the graph Laplacian, Ψ and Λ denote its eigenvectors and eigenvalues, respectively, and h(Λ) defines a filter function in the spectral domain. This graph-based approach enables smooth, adaptive density estimation over complex biological manifolds, providing a principled way to measure how perturbations affect the distribution of cellular states.

**Distribution comparison of different traits.** We quantify the effects of experimental perturbations at single-cell resolution by systematically analyzing the variation across biological replicates within each experimental group and condition. Specifically, we first assessed the variability between replicates within each genotype-treatment combination WT-Veh, WT-KA3, WT-KA25, KO-Veh, KO-KA3 and KO-KA25 to ensure the consistency and reliability of our experimental data.

Next, we characterized the distribution of cellular responses for each genotype-treatment group using kernel density estimation (KDE), a non-parametric approach that provides a smooth estimate of the underlying probability density function without assuming a specific parametric form. KDE allowed us to visualize and quantify the distributional differences and similarities across experimental conditions, thereby providing a robust statistical framework for comparing cellular responses at the population level. To quantitatively assess similarity between probability distributions from different experimental conditions, we use the 1-Wasserstein distance (a.k.a. Earth Mover’s Distance) [94]. This geometry-aware metric measures the minimal “cost” of transforming one distribution into the other and is well suited to biological data where shifts in location and spread are meaningful.

Given two probability distributions *P* and *Q* on ℝ with cumulative distribution functions $F_{P}$ and $F_{Q}$, the 1-Wasserstein distance is


$$\begin{array}{c} W_{1}(P,Q)=\int_{-\infty }^{\infty }|F_{P}(x)-F_{Q}(x)|\;dx, \end{array}$$

or equivalently,


$$\begin{array}{c} W_{1}(P,Q)=\underset{\gamma \in \Gamma (P,Q)}{\inf}\int |x-y|\;d\gamma (x,y), \end{array}$$

where Γ(P,Q) is the set of all couplings with marginals *P* and *Q*. The distance satisfies *W*_1_(*P*, *Q*) = 0 if and only if the distributions are identical. The value is expressed in the units of the variable (e.g., expression) and is bounded by the width of the common support: if both distributions lie in [*a*,*b*], then $0\leq W_{1}(P,Q)\leq b-a$. For comparability across features, we report a normalized Wasserstein distance


$$\begin{array}{c} W_{1}^{*}(P,Q)=\frac{W_{1}(P,Q)}{b-a}\in [0,1], \end{array}$$

so that values near 0 indicate very similar distributions and values closer to 1 indicate large differences. This metric’s sensitivity to both location and shape differences, along with its clear physical interpretation, makes it well-suited for comparing distributions derived from single-cell analyses. Because this analysis pools nuclei within each genotype–treatment group, it does not treat the mouse as the unit of replication, and we present the resulting distances as a descriptive ranking rather than as a hypothesis test.

## Supporting information

S1 Appendix S1 Appendix file (single Word document). (DOCX)
