# Supplementary material for: Cd47 deficiency in mouse brain under excitotoxic stress
Source: PLoS Comput Biol. 2026 Oct 29;22(10):e1014897. doi: 10.1371/journal.pcbi.1014897 (PMC13734605; doi:10.1371/journal.pcbi.1014897)
Supplement: Supplementary file 1 [file pcbi.1014897.s001.docx]

**Supplementary Materials**

Cd47 Deficiency in Mouse Brain Under Excitotoxic Stress

Mehrdad Kiamari^1^*^,^*^2,^*, Amalia Perna^1^, Matteo Santoro^1^, Abdel El Ariss^2^, Eloise Berson^1^*^,^*^2,3,4,5^, Jing Zhao^1^, Chandresh R Gajera^1^, Edward J Fox^1^, Kathleen S Montine^1^, Nima Aghaeepour^2^, and Thomas J Montine^1^

Affiliations:

^1^Pathology Department, Stanford University School of Medicine, Stanford, California, United States of America

^2^Anesthesia, Peri-operative, and Pain Medicine Department, Stanford University School of Medicine, Stanford, California, United States of America

^3^Mines Paris, PSL Research University, BIO-Centre for Computational Biology, F-75006 Paris, France

^4^Institut Curie, PSL Research University, F-75005 Paris, France

^5^INSERM, U1331, F-75005 Paris, France

*Corresponding author: kiamari@stanford.edu

I. **SUPPLEMENTARY FIGURES: A-O**
II. **SUPPLEMENTARY TABLES: A1–A5, B, C1–C21, D**
III. **SUPPLEMENTARY INFORMATION**
I. **SUPPLEMENTARY FIGURES**

**Fig A**

**Fig B**

**Fig C**

**Fig D**

**Fig E**

**Fig F**

**Fig F.** Pseudobulk differential gene expression analysis comparing Cd47 knockout (KO) and WT mice under vehicle (Veh) treatment. Volcano plots show log₂ fold change versus −log₁₀ adjusted p-value across the analyzed cell types. Created in BioRender. Kiamari, M. (2026) https://core.local.biorender.dev/api/short-link/7rmmbix.

**Fig G**

| **A** |  | | |
| --- | --- | --- | --- |
| **B** |  | | |
| **C** |  | **D** |  |

**Fig G:** Module-trait relationship heatmaps for all neuronal cell types: CA1 (panel **A**) CA3 (**panel B**), Dentate Gyrus (**panel C**), and inhibitory neurons (**panel D**). Each row in the heatmap corresponds to a distinct biological trait, defined by a specific combination of genotype and experimental condition, resulting in six categories: KO-KA25, KO-KA3, KO-Veh, WT-KA25, WT-KA3, and WT-Veh. The numerical values within each cell indicate the correlation coefficient between the module eigengene and the respective genotype-condition trait, with the associated p-values shown in parentheses. The intensity and direction of cell colors reflect the strength and sign of these correlations, enabling easy identification of modules strongly associated with particular genotype-condition combinations. These detailed module–trait relationships provide the underlying data used to identify genotype- and treatment-associated modules summarized in Fig. 3.

**Fig H**

| **A** |  |
| --- | --- |
| **B** |  |
| **C** |  |

**Fig H:** Module-trait relationship heatmaps for all glial cell types: astrocytes (**panel A**), microglia cells (**panel B**), and oligodendrocytes (**panel C**). In the heatmap, each row represents a unique biological condition defined by combining genotype and experimental treatment, resulting in six distinct groups: KO-KA25, KO-KA3, KO-Veh, WT-KA25, WT-KA3, and WT-Veh. Numbers displayed within each cell represent the correlation coefficients between the module eigengene and corresponding genotype-treatment condition, with p-values indicated in parentheses. The color intensity and shading direction illustrate the magnitude and sign of these correlations. These detailed module–trait relationships provide the underlying data used to identify genotype- and treatment-associated modules summarized in Fig. 3.

**Fig I**

**Fig I: Treatment Effects on Module-Trait Correlations.** Fig. displays statistically significant (p < 0.05) changes in module-trait correlations (Δr) following treatment with Kainic Acid. The plot shows the change in correlation for low-dose (KA3) and high-dose (KA25) contrasts within both WT (blue) and KO (red) genotypes. Correlation changes with an absolute magnitude below 0.2 are colored grey, and modules are sorted with the strongest responders at the bottom.

**Fig J**

**Fig J: Cross-module analysis identifies highly overlapping genotype- or drug-responsive modules with Jaccard similarity score >= 0.05.** (**A**) Comparison of modules across different neuronal cell types (CA1, CA3, DG, and inhibitory neurons) using the Jaccard similarity metric greater or equal to 0.05. The values on the edges represent the Jaccard similarity scores. Different colors indicate different neuronal cell types. **(B)** Comparison of modules across different glial cell types (astrocytes, oligodendrocytes, and microglia) using the Jaccard similarity metric greater or equal to 0.05. The values on the edges represent the Jaccard similarity scores. Different colors indicate different glial cell types. (**C-D**) Bubble plots showing the overlap of co-expressed gene modules identified in **(C)** different neuronal cell types and **(D)** different glial cell types. In each panel, a circle represents the intersection between a module from one cell type (y-axis) and another (x-axis). The size of the circle is proportional to the Jaccard similarity score, reflecting the fraction of shared genes, while the color indicates the statistical significance (p-value) of the overlap. Non-white colors highlight statistically significant intersections.

**Fig K**

**Fig K:** Enrichment analysis for the remaining modules associated with the CA1 and CA3 regions, including CA1-3, CA1-7, CA3-9, and CA3-16 using GO, KEGG, WikiPathways, REACTOME, and SynGO databases. The size of each circle reflects the proportion of genes linked to the corresponding term, while the color of each circle indicates the statistical significance, represented as $-log \mathrm{FDR}$, where FDR is the false discovery rate.

**Fig L**

**Fig L:** Enrichment analysis for the remaining modules associated with the Inhibitory region, including Inhibitory-2, Inhibitory-6, Inhibitory-7, Inhibitory-11, Inhibitory-14, Inhibitory-18, and Inhibitory-22 using GO, KEGG, WikiPathways, REACTOME, and SynGO databases. The size of each circle reflects the proportion of genes linked to the corresponding term, while the color of each circle indicates the statistical significance, represented as -$log \mathrm{FDR}$, where $FDR$ is the false discovery rate.

**Fig M**

**Fig N**

| **A** |  | **B** |  |
| --- | --- | --- | --- |
| **C** |  | **D** |  |
| **E** |  |  |  |

**Fig N:** Co-expression network of the top 20 genes in modules Inhibitory-1 (**panel A**), DG-1 (**panel B**), Astro-1 (**panel C**), Microglia-9 (**panel D**), and Oligo-1 (**panel E**) based on degree centrality. Each node represents a gene, and edges indicate weighted co-expression strength between gene pairs, derived from soft-thresholded correlations of their expression profiles across samples. Degree centrality measures how broadly a gene's expression co-varies with other module members; it does not measure transcriptional regulation.

**Fig O**

**Fig O: Phenotypic summary of kainic acid (KA) intraperitoneal administration at a sub-convulsive (3 mg/kg) and convulsive (25 mg/kg) dose.** All experiments were conducted in accordance with Stanford University APLAC guidelines (Protocol #31890). Mice received a single intraperitoneal injection of KA (Millipore-Sigma, 420318) dissolved in 0.9% saline (10 ml/kg) and were immediately placed in an open-field arena (40 × 40 cm) for video recording and behavioral tracking using the ANY-MAZE system. Seizure severity was scored every 15 min using a modified Racine scale. (**A**) Comparable body weights across treatment groups. (**B**) Average Racine scale score over the first hour was significantly elevated in the KA 25 mg/kg group compared with Vehicle and KA 3 mg/kg groups (Kruskal–Wallis test, H = 9.415, p = 0.009). Average locomotor activity parameters measured during the first hour following KA administration. (**C**) Path length and (**D**) average locomotor speed were both significantly reduced in KA 25 mg/kg–treated animals relative to Vehicle and KA 3 mg/kg groups. (**E**) Freezing episode counts were significantly increased following KA 25 mg/kg treatment compared with controls. Behavioral locomotor parameters were analyzed using one-way ANOVA followed by Dunnet post-hoc pairwise analysis to the vehicle group. Data are shown as mean ± SEM with individual data points; *p < 0.05, **p < 0.01.

II. **SUPPLEMENTARY TABLES**

**Table A1.** Library-level sequencing and alignment metrics generated by Cell Ranger 7.0.1, with the mapping between Cell Ranger library identifiers and the mouse identifiers used throughout the manuscript.

| **Library ID** | **Mouse ID** | **Sex** | **Treatment** | **Genotype** | **Number of reads** | **Mean reads per cell** | **Sequencing saturation (%)** | **Valid barcodes (%)** | **Q30 bases, RNA read (%)** | **Reads mapped confidently to genome (%)** | **Reads mapped confidently to transcriptome (%)** | **Fraction reads in cells (%)** | **Cell Ranger estimated cells** | **Median UMI per cell (Cell Ranger)** | **Median genes per cell (Cell Ranger)** | **Total genes detected** |
| --- | --- | --- | --- | --- | --- | --- | --- | --- | --- | --- | --- | --- | --- | --- | --- | --- |
| Veh_ko2 | KO_Veh_1 | M | Veh | KO | 586,762,670 | 22,489 | 37.2 | 96.1 | 94.2 | 91.0 | 55.2 | 51.2 | 26,091 | 2,150 | 1,131 | 26,761 |
| Veh_ko3 | KO_Veh_2 | F | Veh | KO | 86,974,168 | 36,760 | 32.3 | 96.5 | 94.3 | 89.6 | 50.5 | 42.0 | 2,366 | 4,492 | 2,105 | 23,219 |
| Veh_ko4 | KO_Veh_3 | F | Veh | KO | 349,787,524 | 33,891 | 36.3 | 96.6 | 94.1 | 90.5 | 53.4 | 51.9 | 10,321 | 5,011 | 2,314 | 26,086 |
| Veh_wt1 | WT_Veh_1 | M | Veh | WT | 47,234,831 | 33,982 | 32.6 | 97.0 | 93.9 | 90.8 | 59.3 | 39.9 | 1,390 | 4,596 | 2,170 | 21,996 |
| Veh_wt2 | WT_Veh_2 | M | Veh | WT | 382,704,803 | 34,210 | 39.2 | 96.7 | 92.7 | 92.6 | 53.6 | 52.3 | 11,187 | 5,075 | 2,379 | 26,080 |
| Veh_wt3 | WT_Veh_3 | F | Veh | WT | 30,751,548 | 37,320 | 36.4 | 96.6 | 94.5 | 89.1 | 57.6 | 41.5 | 824 | 4,752 | 2,219 | 20,855 |
| KA3_ko1 | KO_KA3_1 | M | KA3 | KO | 78,148,604 | 38,573 | 41.8 | 96.0 | 94.5 | 85.2 | 50.0 | 52.3 | 2,026 | 5,166 | 2,226 | 22,975 |
| KA3_ko2 | KO_KA3_2 | M | KA3 | KO | 520,342,589 | 42,623 | 46.8 | 96.2 | 94.3 | 89.1 | 51.6 | 51.4 | 12,208 | 5,311 | 2,329 | 26,297 |
| KA3_ko4 | KO_KA3_3 | F | KA3 | KO | 141,485,260 | 36,626 | 40.2 | 95.9 | 94.2 | 89.5 | 54.8 | 41.5 | 3,863 | 4,272 | 2,020 | 23,932 |
| KA3_wt1 | WT_KA3_1 | M | KA3 | WT | 273,681,110 | 35,423 | 40.6 | 96.3 | 94.3 | 90.6 | 51.0 | 57.5 | 7,726 | 5,379 | 2,326 | 25,506 |
| KA3_wt2 | WT_KA3_2 | M | KA3 | WT | 450,644,763 | 33,087 | 41.3 | 96.4 | 93.3 | 91.4 | 55.8 | 47.7 | 13,620 | 4,332 | 2,072 | 26,153 |
| KA3_wt3 | WT_KA3_3 | F | KA3 | WT | 375,622,674 | 36,650 | 39.4 | 96.2 | 94.1 | 90.4 | 49.8 | 52.6 | 10,249 | 5,053 | 2,234 | 25,998 |
| KA25_ko2 | KO_KA25_1 | M | KA25 | KO | 320,005,521 | 31,321 | 37.7 | 96.8 | 94.3 | 90.5 | 58.9 | 45.1 | 10,217 | 4,459 | 2,130 | 25,754 |
| KA25_ko4 | KO_KA25_2 | F | KA25 | KO | 71,191,775 | 34,813 | 34.5 | 96.1 | 94.3 | 89.6 | 51.2 | 52.2 | 2,045 | 5,393 | 2,322 | 23,211 |
| KA25_wt4 | WT_KA25_1 | F | KA25 | WT | 404,489,441 | 35,225 | 36.0 | 96.2 | 94.4 | 89.9 | 50.4 | 55.0 | 11,483 | 5,457 | 2,447 | 26,375 |

*Cell Ranger cell calling was not used downstream. Nuclei were called from the raw feature-barcode matrix using the filters described in Methods, so "Cell Ranger estimated cells" is a parallel estimate rather than a stage in the analysis pipeline. Read-level metrics are library-level outputs and cannot be attributed to individual nuclei or cell types.*

**Table A2.** Per-mouse nuclei recovery, annotation rate and per-nucleus sequencing depth after quality control.

| **Treatment** | **Genotype** | **Mouse ID** | **Nuclei post-QC** | **Nuclei annotated** | **Annotated (%)** | **Median UMI per nucleus** | **Mean UMI per nucleus** | **Median genes per nucleus** | **Mean genes per nucleus** | **Median mitochondrial counts (%)** |
| --- | --- | --- | --- | --- | --- | --- | --- | --- | --- | --- |
| Veh | KO | KO_Veh_1 | 7,097 | 7,097 | 100.0 | 5,536 | 6,228 | 2,508 | 2,489 | 1.91 |
| Veh | KO | KO_Veh_2 | 1,651 | 1,651 | 100.0 | 3,879 | 4,800 | 1,902 | 1,942 | 0.34 |
| Veh | KO | KO_Veh_3 | 5,962 | 5,962 | 100.0 | 5,167 | 5,824 | 2,347 | 2,324 | 1.10 |
| Veh | WT | WT_Veh_1 | 816 | 816 | 100.0 | 3,617 | 4,645 | 1,818 | 1,899 | 0.40 |
| Veh | WT | WT_Veh_2 | 7,295 | 7,295 | 100.0 | 5,438 | 6,129 | 2,503 | 2,457 | 1.43 |
| Veh | WT | WT_Veh_3 | 571 | 571 | 100.0 | 3,863 | 4,629 | 1,910 | 1,926 | 0.27 |
| KA3 | KO | KO_KA3_1 | 1,322 | 1,322 | 100.0 | 4,376 | 4,970 | 2,005 | 1,959 | 0.27 |
| KA3 | KO | KO_KA3_2 | 7,071 | 7,071 | 100.0 | 4,935 | 5,620 | 2,235 | 2,231 | 1.09 |
| KA3 | KO | KO_KA3_3 | 2,585 | 2,585 | 100.0 | 3,566 | 4,363 | 1,792 | 1,820 | 0.67 |
| KA3 | WT | WT_KA3_1 | 4,528 | 4,528 | 100.0 | 4,716 | 5,289 | 2,120 | 2,083 | 0.54 |
| KA3 | WT | WT_KA3_2 | 6,451 | 6,451 | 100.0 | 4,937 | 5,551 | 2,248 | 2,246 | 1.61 |
| KA3 | WT | WT_KA3_3 | 6,561 | 6,561 | 100.0 | 4,432 | 5,247 | 2,055 | 2,075 | 0.99 |
| KA25 | KO | KO_KA25_1 | 4,720 | 4,720 | 100.0 | 4,784 | 5,395 | 2,234 | 2,267 | 1.55 |
| KA25 | KO | KO_KA25_2 | 1,247 | 1,247 | 100.0 | 3,910 | 5,056 | 1,868 | 1,974 | 0.27 |
| KA25 | WT | WT_KA25_1 | 6,692 | 6,692 | 100.0 | 5,296 | 5,876 | 2,383 | 2,347 | 0.71 |

*Depth was recomputed from the pseudobulk counts layer and therefore excludes mitochondrial genes. All nuclei passing quality control were assigned to one of the seven analysed cell types.*

**Table A3.** Nuclei recovered per mouse and per annotated cell type.

| **Treatment** | **Genotype** | **Mouse ID** | **Astro** | **CA1** | **CA3** | **DG** | **Inhibitory** | **Microglia** | **Oligo** | **Total annotated** | **Total post-QC** |
| --- | --- | --- | --- | --- | --- | --- | --- | --- | --- | --- | --- |
| Veh | KO | KO_Veh_1 | 528 | 1,276 | 790 | 1,951 | 1,054 | 296 | 1,202 | 7,097 | 7,097 |
| Veh | KO | KO_Veh_2 | 171 | 264 | 149 | 410 | 172 | 91 | 394 | 1,651 | 1,651 |
| Veh | KO | KO_Veh_3 | 477 | 1,057 | 545 | 1,860 | 632 | 254 | 1,137 | 5,962 | 5,962 |
| Veh | WT | WT_Veh_1 | 81 | 147 | 62 | 182 | 89 | 50 | 205 | 816 | 816 |
| Veh | WT | WT_Veh_2 | 381 | 1,538 | 695 | 2,445 | 601 | 258 | 1,377 | 7,295 | 7,295 |
| Veh | WT | WT_Veh_3 | 73 | 92 | 45 | 159 | 48 | 27 | 127 | 571 | 571 |
| KA3 | KO | KO_KA3_1 | 126 | 229 | 121 | 338 | 154 | 66 | 288 | 1,322 | 1,322 |
| KA3 | KO | KO_KA3_2 | 529 | 1,247 | 728 | 1,980 | 937 | 440 | 1,210 | 7,071 | 7,071 |
| KA3 | KO | KO_KA3_3 | 255 | 398 | 207 | 617 | 301 | 172 | 635 | 2,585 | 2,585 |
| KA3 | WT | WT_KA3_1 | 524 | 688 | 365 | 1,289 | 614 | 238 | 810 | 4,528 | 4,528 |
| KA3 | WT | WT_KA3_2 | 506 | 1,255 | 611 | 1,896 | 864 | 201 | 1,118 | 6,451 | 6,451 |
| KA3 | WT | WT_KA3_3 | 568 | 1,063 | 582 | 1,967 | 725 | 447 | 1,209 | 6,561 | 6,561 |
| KA25 | KO | KO_KA25_1 | 356 | 917 | 545 | 1,234 | 751 | 128 | 789 | 4,720 | 4,720 |
| KA25 | KO | KO_KA25_2 | 130 | 166 | 79 | 345 | 128 | 70 | 329 | 1,247 | 1,247 |
| KA25 | WT | WT_KA25_1 | 443 | 1,264 | 641 | 1,737 | 891 | 348 | 1,368 | 6,692 | 6,692 |
| **All** | **All** | **All** | **5,148** | **11,601** | **6,165** | **18,410** | **7,961** | **3,086** | **12,198** | **64,569** | **64,569** |

**Table A4.** Within-cell-type sequencing depth per mouse. (i) Median UMI counts per nucleus.

| **Treatment** | **Genotype** | **Mouse ID** | **Astro** | **CA1** | **CA3** | **DG** | **Inhibitory** | **Microglia** | **Oligo** |
| --- | --- | --- | --- | --- | --- | --- | --- | --- | --- |
| Veh | KO | KO_Veh_1 | 2,660 | 7,879 | 10,901 | 5,767 | 6,492 | 1,727 | 2,247 |
| Veh | KO | KO_Veh_2 | 2,028 | 7,035 | 10,249 | 5,190 | 6,001 | 1,152 | 1,476 |
| Veh | KO | KO_Veh_3 | 2,503 | 7,722 | 10,265 | 5,478 | 6,845 | 1,544 | 2,025 |
| Veh | WT | WT_Veh_1 | 1,706 | 6,733 | 10,486 | 4,651 | 5,491 | 1,010 | 1,367 |
| Veh | WT | WT_Veh_2 | 2,211 | 7,807 | 11,505 | 5,557 | 7,089 | 1,455 | 1,902 |
| Veh | WT | WT_Veh_3 | 1,677 | 6,744 | 10,019 | 4,508 | 5,846 | 887 | 1,435 |
| KA3 | KO | KO_KA3_1 | 1,739 | 7,363 | 8,966 | 4,980 | 5,264 | 1,074 | 1,401 |
| KA3 | KO | KO_KA3_2 | 2,112 | 7,299 | 10,137 | 5,308 | 6,158 | 1,572 | 1,979 |
| KA3 | KO | KO_KA3_3 | 1,715 | 6,304 | 9,285 | 4,677 | 5,180 | 1,115 | 1,388 |
| KA3 | WT | WT_KA3_1 | 2,138 | 7,512 | 9,274 | 5,123 | 6,540 | 1,282 | 1,688 |
| KA3 | WT | WT_KA3_2 | 2,207 | 7,110 | 9,709 | 5,012 | 6,008 | 1,663 | 1,866 |
| KA3 | WT | WT_KA3_3 | 2,024 | 7,460 | 10,132 | 4,889 | 6,159 | 1,401 | 1,700 |
| KA25 | KO | KO_KA25_1 | 2,260 | 6,571 | 8,945 | 4,754 | 5,754 | 1,470 | 1,761 |
| KA25 | KO | KO_KA25_2 | 2,008 | 8,403 | 13,146 | 5,338 | 6,866 | 1,150 | 1,353 |
| KA25 | WT | WT_KA25_1 | 2,265 | 7,946 | 10,500 | 5,922 | 6,407 | 1,464 | 1,800 |

**Table A4. (continued).** (ii) Median number of genes detected per nucleus.

| **Treatment** | **Genotype** | **Mouse ID** | **Astro** | **CA1** | **CA3** | **DG** | **Inhibitory** | **Microglia** | **Oligo** |
| --- | --- | --- | --- | --- | --- | --- | --- | --- | --- |
| Veh | KO | KO_Veh_1 | 1,506 | 3,024 | 3,648 | 2,564 | 2,796 | 1,194 | 1,323 |
| Veh | KO | KO_Veh_2 | 1,164 | 2,779 | 3,435 | 2,278 | 2,542 | 814 | 870 |
| Veh | KO | KO_Veh_3 | 1,407 | 3,011 | 3,516 | 2,424 | 2,846 | 1,098 | 1,185 |
| Veh | WT | WT_Veh_1 | 1,038 | 2,734 | 3,534 | 2,173 | 2,413 | 770 | 830 |
| Veh | WT | WT_Veh_2 | 1,276 | 3,128 | 3,843 | 2,532 | 2,958 | 1,044 | 1,106 |
| Veh | WT | WT_Veh_3 | 975 | 2,754 | 3,436 | 2,106 | 2,552 | 691 | 840 |
| KA3 | KO | KO_KA3_1 | 1,014 | 2,726 | 3,154 | 2,196 | 2,291 | 784 | 816 |
| KA3 | KO | KO_KA3_2 | 1,245 | 2,834 | 3,398 | 2,326 | 2,587 | 1,115 | 1,157 |
| KA3 | KO | KO_KA3_3 | 990 | 2,555 | 3,191 | 2,114 | 2,312 | 808 | 814 |
| KA3 | WT | WT_KA3_1 | 1,202 | 2,810 | 3,195 | 2,242 | 2,598 | 919 | 977 |
| KA3 | WT | WT_KA3_2 | 1,268 | 2,832 | 3,359 | 2,258 | 2,577 | 1,152 | 1,110 |
| KA3 | WT | WT_KA3_3 | 1,176 | 2,783 | 3,376 | 2,173 | 2,561 | 989 | 1,012 |
| KA25 | KO | KO_KA25_1 | 1,315 | 2,804 | 3,354 | 2,190 | 2,574 | 1,071 | 1,084 |
| KA25 | KO | KO_KA25_2 | 1,140 | 3,046 | 4,010 | 2,287 | 2,680 | 840 | 818 |
| KA25 | WT | WT_KA25_1 | 1,300 | 3,142 | 3,694 | 2,501 | 2,710 | 1,046 | 1,052 |

**Table A5.** Nuclei counts and per-nucleus sequencing depth per cell type, genotype and treatment group.

| **Cell type** | **Treatment** | **Genotype** | **Mice (n)** | **Nuclei (n)** | **Minimum nuclei per mouse** | **Median UMI per nucleus** | **Median genes per nucleus** |
| --- | --- | --- | --- | --- | --- | --- | --- |
| Astro | Veh | KO | 3 | 1,176 | 171 | 2,510 | 1,416 |
| Astro | Veh | WT | 3 | 535 | 73 | 1,994 | 1,185 |
| Astro | KA3 | KO | 3 | 910 | 126 | 1,930 | 1,148 |
| Astro | KA3 | WT | 3 | 1,598 | 506 | 2,136 | 1,215 |
| Astro | KA25 | KO | 2 | 486 | 130 | 2,189 | 1,264 |
| Astro | KA25 | WT | 1 | 443 | 443 | 2,265 | 1,300 |
| CA1 | Veh | KO | 3 | 2,597 | 264 | 7,712 | 3,002 |
| CA1 | Veh | WT | 3 | 1,777 | 92 | 7,633 | 3,068 |
| CA1 | KA3 | KO | 3 | 1,874 | 229 | 7,011 | 2,760 |
| CA1 | KA3 | WT | 3 | 3,006 | 688 | 7,337 | 2,811 |
| CA1 | KA25 | KO | 2 | 1,083 | 166 | 6,791 | 2,840 |
| CA1 | KA25 | WT | 1 | 1,264 | 1,264 | 7,946 | 3,142 |
| CA3 | Veh | KO | 3 | 1,484 | 149 | 10,555 | 3,600 |
| CA3 | Veh | WT | 3 | 802 | 45 | 11,307 | 3,804 |
| CA3 | KA3 | KO | 3 | 1,056 | 121 | 9,786 | 3,343 |
| CA3 | KA3 | WT | 3 | 1,558 | 365 | 9,808 | 3,336 |
| CA3 | KA25 | KO | 2 | 624 | 79 | 9,297 | 3,426 |
| CA3 | KA25 | WT | 1 | 641 | 641 | 10,500 | 3,694 |
| DG | Veh | KO | 3 | 4,221 | 410 | 5,555 | 2,483 |
| DG | Veh | WT | 3 | 2,786 | 159 | 5,432 | 2,491 |
| DG | KA3 | KO | 3 | 2,935 | 338 | 5,107 | 2,258 |
| DG | KA3 | WT | 3 | 5,152 | 1,289 | 5,006 | 2,226 |
| DG | KA25 | KO | 2 | 1,579 | 345 | 4,835 | 2,214 |
| DG | KA25 | WT | 1 | 1,737 | 1,737 | 5,922 | 2,501 |
| Inhibitory | Veh | KO | 3 | 1,858 | 172 | 6,559 | 2,790 |
| Inhibitory | Veh | WT | 3 | 738 | 48 | 6,868 | 2,902 |
| Inhibitory | KA3 | KO | 3 | 1,392 | 154 | 5,863 | 2,488 |
| Inhibitory | KA3 | WT | 3 | 2,203 | 614 | 6,210 | 2,573 |
| Inhibitory | KA25 | KO | 2 | 879 | 128 | 5,867 | 2,576 |
| Inhibitory | KA25 | WT | 1 | 891 | 891 | 6,407 | 2,710 |
| Microglia | Veh | KO | 3 | 641 | 91 | 1,566 | 1,118 |
| Microglia | Veh | WT | 3 | 335 | 27 | 1,353 | 981 |
| Microglia | KA3 | KO | 3 | 678 | 66 | 1,378 | 1,000 |
| Microglia | KA3 | WT | 3 | 886 | 201 | 1,404 | 1,000 |
| Microglia | KA25 | KO | 2 | 198 | 70 | 1,384 | 1,008 |
| Microglia | KA25 | WT | 1 | 348 | 348 | 1,464 | 1,046 |
| Oligo | Veh | KO | 3 | 2,733 | 394 | 2,029 | 1,197 |
| Oligo | Veh | WT | 3 | 1,709 | 127 | 1,823 | 1,061 |
| Oligo | KA3 | KO | 3 | 2,133 | 288 | 1,712 | 1,003 |
| Oligo | KA3 | WT | 3 | 3,137 | 810 | 1,761 | 1,040 |
| Oligo | KA25 | KO | 2 | 1,118 | 329 | 1,666 | 1,012 |
| Oligo | KA25 | WT | 1 | 1,368 | 1,368 | 1,800 | 1,052 |

*Nuclei counts are those entering the analysis before genotype balancing. Within each treatment condition, KO and WT nuclei were downsampled to the smaller group before co-expression network construction.*

**Table B: Summary of WGCNA module membership counts.** Total number of genes (Gene ct.) assigned to each co-expression module (Mod.) across neuronal (CA1, CA3, DG, Inhibitory) and glial (Astrocyte, Microglia, Oligodendrocyte) populations. Module nomenclature (e.g., CA1-1) reflects the hippocampal subregion/cell type and the specific module index assigned during network construction.

| **Mod.** | CA1-  1 | CA1-  2 | CA1-  3 | CA1-  6 | CA1-  9 | DG-  1 | DG-  12 | Inhib-  1 | Inhib-  6 | Inhib-  18 | Inhib-  22 | Astro-  1 | Micro-  1 | Micro-  2 | Micro-  9 | Oligo-  1 | Oligo-  2 | Oligo-  12 |
| --- | --- | --- | --- | --- | --- | --- | --- | --- | --- | --- | --- | --- | --- | --- | --- | --- | --- | --- |
| **Genect.** | 409 | 975 | 3,664 | 15,225 | 78 | 10,804 | 13,302 | 12,762 | 2,475 | 1,120 | 1,531 | 15,645 | 4,210 | 2,588 | 7,549 | 12,817 | 2,096 | 6,173 |

| **Mod.** | CA3-  1 | CA3-  2 | CA3-  5 | CA3-  6 | CA3-  8 | CA3-  9 | CA3-  10 | CA3-  12 | CA3-  13 | CA3-  14 | CA3-  16 | CA3-  18 | CA3-  21 | CA3-  22 | CA3-  24 | CA3-  29 | CA3-  36 | CA3-  38 | CA3-  40 |
| --- | --- | --- | --- | --- | --- | --- | --- | --- | --- | --- | --- | --- | --- | --- | --- | --- | --- | --- | --- |
| **Genect.** | 325 | 6,220 | 65 | 3,806 | 94 | 306 | 227 | 155 | 129 | 76 | 1,921 | 2,021 | 1,369 | 78 | 115 | 98 | 81 | 61 | 109 |

**Table C: Top differentially expressed genes for all regions.**

| **Table C1*.*** Top differentially expressed genes in **hippocampal microglia**. List of significant genes exhibiting the largest expression change ($\vert log Fold Change\vert>=0.5$) in Cd47 KO compared to WT under **vehicle** condition. | |
| --- | --- |
| **Upregulated**  **genes** | *Apoe, Arhgap31, Mt1, Ptgds, Camk1d, Cox8a, Meg3, Calm1, AY036118, Clu, Atp6v0c* |
| **Downregulated**  **genes** | *Entpd1, Frmd4a, C1qa, Zfhx3, Ccnd3, Selplg, Ttr* |

| **Table C2*.*** Top differentially expressed genes in **hippocampal CA3**. List of significant genes exhibiting the largest expression change ($\vert log Fold Change\vert>=0.5$) in Cd47 KO compared to WT under **vehicle** condition. | |
| --- | --- |
| **Upregulated**  **genes** | *AC149090.1, Cst3, Gm42418, Apoe, Kcnj3, Tmeff2, Mt1, Rplp1, Ptgds, Negr1, Mt3, Dbi, Dynlt1a, Lin7a, Sparcl1, B230209E15Rik, Rps29, Htr4, Rpl34, Kirrel3, Tenm1, Sst, Rps26, Plp1, Ptn, Rpl35a, Trpm3, Uqcr11, Etv1, Gm15577, Cntn4, Aldoc, Nrg1, Clu, Rpl13a, Rpl36, Pdia5, Rps23, Rpl23a, Dynlt1f, Rpl39, Gm21954, Atp5e, Atp1a2, Rpl27, Filip1l, Gm15594, Lzts1, Ptprz1, 4930419G24Rik* |
| **Downregulated**  **genes** | *Actl6a, Irf3, Mir22hg, Mib2, Fndc5, Klf10, Ackr3, Zfp27, Cebpg, Wrap73, Gm16159, Ctsb, Tcta, Bloc1s2, Alkbh5, Fcf1, Mfsd13a, Gm10851, Gls2, Rab5if, Zcchc9, Gm11508, Pop5, Cnbp, Gfra4, Arpc5l, Gm10501, Arc, Sap18, Smad7, Gm31323, Syngr3, Jpt1, Tent4a, Srp14, Zbtb1, Lrrc57, Fbxo33, Rhof, Stac2, Ccdc107, Chst2, Arvcf, Akirin1, Nsmce3, Trub2, Alg2, Brms1, Eno1, Thap2, Gm12027, Tmem9b, Gm27003, 5430400D12Rik, Il34, AC163685.1, Tiprl, 0610043K17Rik, 0610030E20Rik, Pgam5, Gm35501, Gal3st3, Slco2a1, Neu1, Gm15965, Vps37d, Mfsd11, Emc3, Trnp1, Dcakd, 2300009A05Rik, Borcs5, Gm38134, Ppp1r10, Tmem179, Zbtb18, Khsrp, Tesc, Islr2, Npy2r, Gpr18, Prr14, Skida1, Pcgf6, Dhps, Gm16638, Klhl26, Zmym1, Gm14703, Jkamp, Lrrc10b, Lats2, Letm2, Slc25a51, Cox19, Gm48003, Ajap1, Ly6e, E130102H24Rik, Thoc7, Pfn2, Arpc5, Ogfrl1, Napepld, Lyn, Smoc2, Snhg20, 4930578M01Rik, Caml, Pcyt2, Pla2g3, Scn1b, Mllt11, Mri1, Zfp329, Homer3, Pebp1, Mgll, Npdc1, Gm10516, Cenpv, Ndufaf4, Qtrt1, 2610035D17Rik, Ywhah, Mdga1, Rnps1, Kctd4, Neurod6, Taf1d, Gm27032, Wscd2, Gm44257, Srsf7, Chgb, Gm47283, Acp1, Ttr* |

| **Table C3.** Top differentially expressed genes in **inhibitory neurons**. List of significant genes exhibiting the largest expression change ($\vert log Fold Change\vert>=0.5$) in Cd47 KO compared to WT under **vehicle** condition. | |
| --- | --- |
| **Upregulated**  **genes** | *Cst3, Apoe, AC149090.1, Ptgds, Mt1, Camk1d, Fth1, Rplp1, Cntnap2, Mt3, Cox6c, Slc1a2, Tmsb4x, Rps29, Rps21, Rpl37, Cox8a, Rpl38, Rpl26, Clu, Ppia, Rpl41, Cntn5, Rpl39, Ptn, Plp1, Rps8, Rps20, Rps25, Dbi, Rpl37a, Rpl23, Rps23, Rpl21, Rpl30, Rpl19, Rps24, Rps26, Fau, Rps14, Dcc, Aldoa, Rps27a, Cmss1, Rpl34, Cox5b, Aldoc, Rplp2, Slc17a7, Rps19, Sst, Rps10, Mbp, Uqcr11, Atp5e, Atp5mpl, Ndrg2, Rps16, Rpl12, Rps28, Rpl28, Rpl36, Scd2, Ndn, Camk2n1, Rps17, Cox6b1, Chchd2, Gm28379, Rpl32, Rps4x, Atp5k, Ndufa13, Ndufa2, Pde1a, Gm26854* |
| **Downregulated**  **genes** | *Dazap1, Sh3pxd2a, Gm14204, Pla2g3, Stac2, Psmd9, Gm14703, 5031415H12Rik, Rnf34, Gm11418, Pag1, Sgsm3, Gm38134, Timmdc1, Gm15478, 2610035D17Rik, Acp1, Lyn, Plekhm2, Macrod1, Slc32a1, Rab40c, Mgll, Cacng8, Gm27032, A330102I10Rik, Rbm4b, Srsf7, Prickle1, Gm47283, Ttr* |

| **Table C4.** Top differentially expressed genes in **oligodendrocytes**. List of significant genes exhibiting the largest expression change ($\vert log Fold Change\vert>=0.5$) in Cd47 KO compared to WT under **vehicle** condition. | |
| --- | --- |
| **Upregulated**  **genes** | *Cst3, Camk1d, Apoe, Meg3, AY036118, AC149090.1, Mylk, Ctnna2, Syt1, Gpm6a, Mt1, Lsamp, Slc1a2, Cox8a, Cpe, Sparcl1, Rplp1, Atp1b1, Nrxn1, Mt3, Pcdh11x, Map2, Rpl38, Pcsk1n, Snap25, Clu, Negr1, Nme7, Cmss1, Sst, Csmd1, Cox6c, Astn2, Dmd, Dpp10, Map1b, Cox7b, Nrg3, Gria2, Rpl37, Rps26, Tspan7, Olfm1, Celf2, Rbfox1, Rims2, Plcb1, Dpp6, Nrxn3, Brinp1, Ccser1, Atp1a3, Fam155a, Ppp2r2b, Ly6h, Dlgap1, Rora, Lhfpl3, Cox7a2, Kcnma1, Dock4, Camk2a, Gria3, Rps12, Ntm, Kcnd2, Rpl41, Rps29, Syn2, Rps15, Dpyd, Adgrb3, Rpl37a, Selenow, Atp5md, Fgf14, Psap, Ntrk2, Rpl35a, Basp1, Lrrtm4, Grm5, Atp1a2, Dbi, Pcdh15, Ctnnd2, Ndufc2, Ptprz1, Camk2n1, Dscam, Rims1, Gapdh, Tnr, Pcdh7, Snhg11, Adgrl3, Rps23, Nav3, Atpif1, Kcnip4, Atp2a2, Gpc5, Brinp3, Sntg1, Arpp21, Cntn1, Ptprt, Atp5e, C4b, Grid1, Luzp2, Zfpm2, Sgcd, Ndufs5, Rpl26, 1700111E14Rik, Uqcr11, Slc35f1, Rpl32, Gm4876, Mmp16, Pja2, Hivep2, Lrrc7, Adarb2, Pcp4, Kalrn, Cntn4, Rps19, Sgcz, Nnat, Nsg2, Inpp4b, Thy1, Tenm2, Slc17a7, Erc2, Zeb1, Dclk1, Prkg1, 2900097C17Rik, Uqcr10, Galnt13, Atp5j, Gpc6, Cox6b1, Aldoc, Chn1* |
| **Downregulated**  **genes** | *Pla2g3, Gal3st1, Cpm, Ptpn11, Kctd13, Smad7, Rhou, C030029H02Rik, Clmn, Ttyh2, Sept4, Tnfaip6, Zdhhc20, Ninj2, Mast3, Aspa, Rnf220, Arhgap23, Adipor2, Vmp1, Fnbp1, Ttr* |

| **Table C5.** Top differentially expressed genes in **hippocampal CA1**. List of significant genes exhibiting the largest expression change ($\vert log Fold Change\vert>=0.5$) in Cd47 KO compared to WT **vehicle** condition. | |
| --- | --- |
| **Upregulated**  **genes** | *AC149090.1, Cst3, Gm42418, Apoe, Mt1, Rplp1, Slc1a2, Ptgds, AY036118, Mt3, Ptn, Dbi, Mlip, Rpl37, Sst, Dynlt1a, Clu, Plp1, Aldoc, Rps26, Scd2, Gm21798, Rpl23a, Rplp2, Rpl39, Ndrg2, Atp5e, Fam13a, Gm15577, Uqcrb, Pcdh10, Atp1a2, Pcp4, Gpc4, Gpx4, Pdia5, Trf, Dynlt1f, Glul, Grb10, Ndn, Sparc, Tuba1b, Ids, Gm41804, Far2os1* |
| **Downregulated**  **genes** | *Man1c1, Olfml2b, Hes1, Gm48855, Tmem158, Eno1, Ccdc107, Tbl3, 4930509H03Rik, Arc, Rasl11b, Rnf167, Gm42556, Tsc22d4, 4732440D04Rik, Gm27003, 4930578M01Rik, Rpl35, Junb, Klhl26, Mri1, Rdh14, Tmed2, Syngr3, Rasl10a, Gpr18, Gm47689, Gm10516, Bcl6, Tesc, Cotl1, Gm12027, Dyrk3, Ppp1r1a, Macrod1, Zmym1, Gm38134, Qtrt1, Dazap1, Ppil2, Gm14703, Gm15965, Doc2b, Pou3f1, Pla2g3, Taf1d, Acp1, Celf3, Gm47283, Srsf7, Ttr* |

| **Table C6.** Top differentially expressed genes in **astrocytes**. List of significant genes exhibiting the largest expression change ($\vert log Fold Change\vert>=0.5$) in Cd47 KO compared to WT under **vehicle** condition. | |
| --- | --- |
| **Upregulated**  **genes** | *Camk1d, Mdga2, Meg3, AY036118, Ptgds, Atp13a4, Cox6c, Asic2, Fth1, Syt1, Rplp1, Astn2, Luzp2, Ccdc85a, Fgf14, Tmsb4x, Olfm1, Kcnd2, Trim9, Atp1a3, Cox4i1, Rpl37, Trpm3, Atp6v0c, Camk2n1, Slc25a3, AC149090.1, Rps29, Il1rapl1, Atp5md, Tuba1a, Rpl37a, Atp1b1, Map1b, Snap25, Sst, Cox7c, Mt3, Rpl23, Pcsk1n, Rtn1, Rps26, Cox5b, Atp5l, Nme7, Cox8a, Elob, Mt1, Tubb2a, Calm1, Nrgn, Nnat, Rpl34, Rpl27a, Rpl26, Nsg2, Rps15, Mfn1, Cntn1, Rpl19, Rpl41, Fam184a, Plp1, Chchd2, Hsp90aa1, Hnrnpu, Ly6h, Etnppl, Hsp90ab1, Rpl30* |
| **Downregulated**  **genes** | *Mgat5, Ttr* |

| **Table C7.** Top differentially expressed genes in **DG neurons**. List of significant genes exhibiting the largest expression change ($\vert log Fold Change\vert>=0.5$) in Cd47 KO compared to WT under **vehicle** condition. | |
| --- | --- |
| **Upregulated**  **genes** | *Gm42418, Cst3, Apoe, AC149090.1, Mt1, Ptgds, Cpe, AY036118, Ptn, Gm15577, Clu, Dbi, Scd2, Cmss1, Sst, Alcam, Dynlt1a, Aldoc, Plp1, Pdia5, Stmn2, Rpl23a, Ndrg2, Ntm, Gm17066, Amy1, Gap43, Atp5e, Gm21954, Tuba1b, Il1rapl2, Atp1a2, Dpp10, Mt2, Hexb, Slc15a2, Trf, Spock1, Ndufc1, Mif, Timp2, Npy, Ndufb7, Hcn1, Filip1l, Glul, Ndn, Ids, Plpp3, S100b* |
| **Downregulated**  **genes** | *Mto1, Gm10516, Mkrn1, Gm27003, Gm15965, Tbl3, Dusp12, Bhlhe22, Cebpb, Lrrc57, Bop1, Sema5b, Gm16286, Babam1, Med6, Tesc, Ntan1, Gm38134, Vash1, Jun, Timmdc1, Qtrt1, Rhobtb2, Pnmal2, B4galt4, C1ql2, Gm12027, Rchy1, Doc2b, Rasl10a, Zmym1, Ppp1r1a, Trim35, Rpl15, Pla2g3, Klf13, Acp1, Srsf7, Ttr* |

| **Table C8.** Top differentially expressed genes in **hippocampal microglia**. List of significant genes exhibiting the largest expression change ($\vert log Fold Change\vert>=0.5$) in Cd47 KO compared to WT under **KA3** condition. | |
| --- | --- |
| **Upregulated**  **genes** | *Arhgap31, Ptprc* |
| **Downregulated**  **genes** | *Gm47283, Ptgds, Fkbp5* |

| **Table C9.** Top differentially expressed genes in **hippocampal CA3**. List of significant genes exhibiting the largest expression change ($\vert log Fold Change\vert>=0.5$) in Cd47 KO compared to WT under **KA3** condition. | |
| --- | --- |
| **Upregulated**  **genes** | *Safb, Pabpc1, Dynlt1a, Rbbp6, Agap2, Ttr, Srsf9, Chpf2, Sag, Tsc1, Prrt1, Tmem181a, Gtf2ird2, Dlk2, Ints3, Gm17066, Samm50, Vxn, CT009536.1, Sfi1, Klhl26, Med23, Esrra, Cetn3, Lonp1, Dynlt1f, Hspd1, Pdia5, Ncln, Park7* |
| **Downregulated**  **genes** | *BC005537, Rpl10, Ubb, 1700111E14Rik, Zmym1, Rpl41, Ptgds, Rpl15, Gm47283, AC149090.1* |

| **Table C10.** Top differentially expressed genes in **inhibitory neurons**. List of significant genes exhibiting the largest expression change ($\vert log Fold Change\vert>=0.5$) in Cd47 KO compared to WT under **KA3** condition. | |
| --- | --- |
| **Upregulated**  **genes** | *Ttr, Pabpc1, Safb, Dynlt1a, Rbbp6, Chpf2, Sfi1, Gm17066, Prrt1, Agap2, Samm50, Sag* |
| **Downregulated**  **genes** | *Rpl10, Rpl15, Rps29, Ubb, Ptgds, Gm47283, AC149090.1* |

| **Table C11.** Top differentially expressed genes in **oligodendrocytes**. List of significant genes exhibiting the largest expression change ($\vert log Fold Change\vert>=0.5$) in Cd47 KO compared to WT under **KA3** condition. | |
| --- | --- |
| **Upregulated**  **genes** | *Mylk, Pabpc1, Safb* |
| **Downregulated**  **genes** | *Spock3, Kctd13, Htra1, Map7d2, Fkbp5, Ephb1, Daam2, Ubb, Bcl2l1, AC149090.1, AY036118, Sgk1, Mast3, Rps29, Zbtb16, Apoe, Gm47283, Adipor2* |

| **Table C12.** Top differentially expressed genes in **hippocampal CA1**. List of significant genes exhibiting the largest expression change ($\vert log Fold Change\vert>=0.5$) in Cd47 KO compared to WT **KA3** condition. | |
| --- | --- |
| **Upregulated**  **genes** | *Safb, Pabpc1, Ttr, Prrt1, Rbbp6, Agap2, Dynlt1a, Grik4, Chpf2, Vxn, Mtrex, Med23, Sag, Srsf9, Gm17066, Esrra, CT009536.1, Sfi1, Lonp1, Flrt1, Samm50, Pdia5, Bc1* |
| **Downregulated**  **genes** | *Gm29237, Mid1, 4930415C11Rik, Rpl10, Rpl35, Ubb, Zmym1, Rpl15, Ptgds, Gm47283, AC149090.1* |

| **Table C13.** Top differentially expressed genes in **astrocytes**. List of significant genes exhibiting the largest expression change ($\vert log Fold Change\vert>=0.5$) in Cd47 KO compared to WT under **KA3** condition. | |
| --- | --- |
| **Upregulated**  **genes** | *Abca1, Paxbp1* |
| **Downregulated**  **genes** | *Rps29, Rin2, Tmcc3, Ccdc85a, Etnppl, Zfhx3, Zbtb16, Chka, AY036118, Nxn, 9330159F19Rik, Sorbs1, Luc7l2, Ubb, Acsl3, Kcnn2, AC149090.1, Cdh19, Rmst, Ptgds* |

| **Table C14.** Top differentially expressed genes in **DG neurons**. List of significant genes exhibiting the largest expression change ($\vert log Fold Change\vert>=0.5$) in Cd47 KO compared to WT under **KA3** condition. | |
| --- | --- |
| **Upregulated**  **genes** | *Grik4, Pabpc1, Safb, Rbbp6, Prrt1, Ttr, Dynlt1a, Srsf9, Agap2, Tenm4, Sag, Chpf2, Thsd7b, Pde1a, Tmem181a, Pdia5, Htr1f, CT009536.1, Klhl26, Gm17066, Kitl, Gm49169, Scn9a, Dlk2, Traip, Hbb-bs, Atp2b4, Hexb, Snrpd3* |
| **Downregulated**  **genes** | *Slc15a2, Gm6999, Stat3, Acvr1c, Rasgrp1, B4galt4, Mid1, Cadps2, Rpl35, Rpl10, Pcdh7, Clmp, Epb41, Syndig1, Zmym1, Brinp3, Rpl15, Ptgds, Pde4d, Unc5d, Ntng1, Gm47283, AC149090.1* |

| **Table C15.** Top differentially expressed genes in **hippocampal microglia**. List of significant genes exhibiting the largest expression change ($\vert log Fold Change\vert>=0.5$) in Cd47 KO compared to WT under **KA25** condition. | |
| --- | --- |
| **Upregulated**  **genes** | *Ttr, Fkbp5* |
| **Downregulated**  **genes** | *Atp6v0c, Cst3, AY036118, Gm42418* |

| **Table C16.** Top differentially expressed genes in **hippocampal CA3**. List of significant genes exhibiting the largest expression change ($\vert log Fold Change\vert>=0.5$) in Cd47 KO compared to WT under **KA25** condition. | |
| --- | --- |
| **Upregulated**  **genes** | *Ttr, 9530059O14Rik, Tmem108, Ssbp4, Tango2, Dgki, Apoe, Chrd, Cachd1, Pid1, Mt1, Kcna1, Rerg, Rap1gap, Jph4, Enpp2, Hes1, Plcl1, Gm44257, Tmem131l, Astn2, Sh3pxd2a, Gna12, 4930578M01Rik, Bcar1, Aatk, Ttll4, Pdia5, Sema5b* |
| **Downregulated**  **genes** | *1700024B18Rik, Gm11149, Psmd12, 3110039M20Rik, Dpp10, Gm39214, Gm2824, Col6a1, 2810432F15Rik, Gm21954, B230209E15Rik, 4930567K12Rik, Inhba, Gm48765, Cd226, Sulf1, Htra2, Cd46, Tmtc4, Brip1os, Gins2, Grip1os2, 4930455C13Rik, Gm15594, 4930512B01Rik, Fndc3a, Ccsap, Gm48747, Dsel, Vegfa, Macrod2os1, D030068K23Rik, Rtl1, Tenm1, Gm10125, Gm28379, Gm47271, Far2, Wsb1, Ice1, Gm26854, Alas2, Mid1, Lrrc40, 4933413L06Rik, Ccdc73, Gm20319, Dnajc19, P2ry12, Tmx4, Gm17066, Spink13, Agfg1, Htr4, Gm9968, Gm16183, Bbof1, Itgbl1, A330008L17Rik, Gm13187, Filip1l, Gm26936, Rreb1, Sorbs2os, Ppib, Gm15414, 4930428E07Rik, Paxbp1, Gm16126, Gm48239, Csmd2os, Gm41505, Ptgds, Hacd4, A230004M16Rik, Hnrnpa2b1, Kansl1l, Nudt5, Gm15680, AY036118, Gm48091, Gm42418, Ahi1, Srrm2, Gm12296, AC149090.1* |

| **Table C17.** Top differentially expressed genes in **inhibitory neurons**. List of significant genes exhibiting the largest expression change ($\vert log Fold Change\vert>=0.5$) in Cd47 KO compared to WT under **KA25** condition. | |
| --- | --- |
| **Upregulated**  **genes** | *Ttr, Mt1, Apoe, Pten, Col19a1, Vstm2b, Zbtb16, Kcna1, Enpp2* |
| **Downregulated**  **genes** | *5530401A14Rik, Dnajc19, Gm16183, Rnf25, Oprm1, Mid1, Filip1l, Ryr3, Gm26749, Fam76b, Gm2516, Gm21954, Far1os, Xrn2, Gm45321, Gm28750, Atg16l2, Kdm6a, Ensa, C130073E24Rik, Ppib, Gm48091, Gabra2, Mettl23, Bbof1, Guf1, Gm47027, Gm37679, Tmx4, Olfm1, Gm15577, Chga, Scg2, Ptgds, Myef2, A230004M16Rik, Gm12296, 4930555F03Rik, Sorbs2os, Wsb1, Hnrnpdl, 4930547E14Rik, Slc24a5, Nudt5, Paxbp1, Actb, Rtl1, Nme7, Gm47283, Hnrnpa2b1, AY036118, Ahi1, Gm42418, AC149090.1* |

| **Table C18.** Top differentially expressed genes in **oligodendrocytes**. List of significant genes exhibiting the largest expression change ($\vert log Fold Change\vert>=0.5$) in Cd47 KO compared to WT under **KA25** condition. | |
| --- | --- |
| **Upregulated**  **genes** | *Ttr, Elmo1, Zbtb16, Mobp, Fkbp5, Kcna1, Mat2a, Adipor2, Sgk1, Bcl2l1, Mt1, Zfand4, Tra2a, Pakap-1, Pakap, Phyhd1, St3gal3* |
| **Downregulated**  **genes** | *Eml1, Mettl23, Gm42413, Sst, Cox6c, Cox8a, Ly6h, Calm2, Cmss1, Rps8, Grm7, Nme7, C030029H02Rik, Rtn1, Rnf220, Hnrnpdl, Mdga2, Olfm1, Atp1b1, Meg3, Srrm2, Cst3, Gm47283, Atp6v0c, Ano4, AC149090.1, Actb, Camk1d, AY036118, Gm42418* |

| **Table C19.** Top differentially expressed genes in **hippocampal CA1**. List of significant genes exhibiting the largest expression change ($\vert log Fold Change\vert>=0.5$) in Cd47 KO compared to WT **KA25** condition. | |
| --- | --- |
| **Upregulated**  **genes** | *Ttr, Dgkg, Pip5k1b, Prkca, Wipf3, Mt1, Fkbp5, Kcnip2, Apoe, Celf3, Stum, Dtx3, Phf20, Itpka, Thrb, Enpp2, Kcnab2, Raver2, Chrd, Ssbp4, Syn3, Rps29, Parp1, Arhgap23, Epha10, Acss1, Gm47591, Adcy1, Mbp, Arel1, Zfhx4, Rhbdl3, Pla2g4e, C230057M02Rik, B4galt1* |
| **Downregulated**  **genes** | *Gm15496, Gm16351, Bche, Gm49417, Gm21798, Calb1, Gm13187, Lhfpl3, Adamtsl1, 4921511I17Rik, 2610203C22Rik, 4930567K20Rik, Clec18a, Gm10135, B3gat2, Pbdc1, Cdh9, Gm28379, Ngly1, Cradd, Epyc, Gm29088, Fam228b, Rph3a, Slit2, Gm17066, 4933433G19Rik, Tmtc4, Lrrc19, Utp18, Ctnna3, Gm16226, Gm47350, Gm10544, Gm45645, 4930567K12Rik, Rwdd1, Eif2s3x, Gm49708, Gm3848, Uvssa, Angpt2, Gm26854, Atp2b4, Alas2, Kif26b, 1700024B18Rik, Gm15398, Tdg, 6030443J06Rik, Fam76b, Med23, Grip1os2, Bms1, Rspo2, Pcdh15, Mettl23, Shisa9, Etv5, Filip1l, Gm14858, Lrrtm4, Socs2, Slc16a4, Gm47448, Smarcd1, Kmt2d, Ccdc73, Ptprk, Vegfa, Gm20319, Nwd2, Gm10558, Zmiz1os1, Lrif1, Atg10, Flrt1, Atg16l2, Usp29, Gm39214, Fgf13, 5530401A14Rik, 4930428E07Rik, Tspan2, Gm44643, Itgb3bp, 4930455C13Rik, Nnat, Frg1, Gm46218, Guf1, Lysmd4, Csmd2os, Cntln, Gm4128, Ccsap, Itgbl1, Gm15577, C630031E19Rik, Inhba, Gm48239, B3galt2, Rftn1, Gm10785, Rtl1, A230004M16Rik, Myo1d, D030068K23Rik, Cbx3, 4930512B01Rik, Gm26749, Usp50, Gm16126, Rpl15, Acvr1c, Fbln2, Megf11, Ptgds, Fndc3a, Gap43, P2ry12, Lin7a, Pabpc1, Ppib, H3f3b, Edil3, Hacd4, 4930532I03Rik, Gab2, Ncam2, Gm19710, Scg2, Grin3a, Rbms3, Rnpc3, Glp2r, Nrg1, Mycbp, Chga, Kdm6a, Ice1, Gm47271, Dsel, Gm26917, 4930509J09Rik, Gm48091, Grik4, Gm15594, 1700111E14Rik, Gm26936, Gm15414, Gm11149, Far1os, Mid1, Macrod2os1, Gm21954, Plxna4os3, A830018L16Rik, 4930547E14Rik, Rreb1, Gm41505, Kansl1l, B230209E15Rik, Actb, Sorbs2os, Hnrnpa2b1, Gm47283, Nudt5, Paxbp1, Gm15680, Gm12296, AY036118, Srrm2, Nrg3os, Gm42418, AC149090.1* |

| **Table C20.** Top differentially expressed genes in **astrocytes**. List of significant genes exhibiting the largest expression change ($\vert log Fold Change\vert>=0.5$) in Cd47 KO compared to WT under **KA25** condition. | |
| --- | --- |
| **Upregulated**  **genes** | *Ttr, Mertk, Rmst, Ttyh1, Abr, Nxn, Zhx3, Ndrg2, Phyhd1, Mt1, Hif3a, Kcnq3, Arhgef4, Paqr8, Asic2, Enpp2, Dcc* |
| **Downregulated**  **genes** | *Ptgds, Cox7c, Specc1, Gm35188, Cpeb4, Actb, Fgf14, Camk1d, Rplp1, Gm47283, Kcnd2, AC149090.1, Srrm2, AY036118, Sat1, Wdr17, Gm42418* |

| **Table C21.** Top differentially expressed genes in **DG neurons**. List of significant genes exhibiting the largest expression change ($\vert log Fold Change\vert>=0.5$) in Cd47 KO compared to WT under **KA25** condition. | |
| --- | --- |
| **Upregulated**  **genes** | *Ttr, Adgrl3, Pdzd2, Rtn4rl1, Ogt, Sntb2, Mt1, Celf3, Fkbp5, Sorcs3, Serinc2, Dot1l, Crtac1, Enpp2, Iqsec3, Nol4l, Fam129b, Snta1, Clmp, Sema3e, Cdyl2, Camkk1, Tesc, Sh2d5, Necab3, Kcnip2* |
| **Downregulated**  **genes** | *Iqcm, Grik3, Gm15283, Atrip, Atp2b4, Notch2, Pde1a, B3gat2, Greb1l, Utp18, Grm3, C630031E19Rik, Vegfa, Gm38604, Hdac5, Ddx41, Atg16l2, Rasgrf2, Nelfa, Gm16183, Fam210a, Mettl23, Etv1, Gm26936, Grin3a, Fat4, Catspere2, 4930512B01Rik, Zfp46, Pbdc1, Gm13544, Mbd4, Pak1, Hacd4, 1700024B18Rik, Txnl1, 4930567K12Rik, Rpl23a, Ncl, Gm30094, Rprml, Tubgcp2, Kmt2d, Gm10558, Gm17066, Vwa3b, Tmem29, Pcsk2os2, Ephb1, Dsel, Emc2, Akap7, Tspyl4, Sec11c, Gda, Rbms1, Gm28379, Gm47271, Frg1, Dcun1d5, A330008L17Rik, Fbxo10, Gm40841, Htra2, Idh3b, F730043M19Rik, Gm37459, Grp, Gm9968, Gm16267, Gm11149, Glp2r, Nckap5, Alcam, Hs3st4, Alas2, Macrod2os1, Me3, D430041D05Rik, Rnpc3, Gm26749, 4833422C13Rik, Lysmd4, Csmd2os, Igsf11, Ppib, Hectd2os, 4930547E14Rik, Gm48091, Tmx4, Gm14858, Gm32014, Ndst3, Flrt3, P2ry12, Fam76b, Gm15680, Calb1, B3galt2, Gm15577, Gm50250, Rps8, Gm48747, Jpx, Dcc, Grid1, Cdh10, Rsrp1, Gucy1a2, Lrrc4, Hnrnpdl, Nme7, Gm21954, A330015K06Rik, Egfem1, Actb, Nudt5, Mid1, Gm43376, Car10, Casc4, Ptgds, Cntnap5b, Hnrnpa2b1, Lca5, Ror1, Gm15594, Sorbs2os, Rreb1, Rpl15, Gm47283, AY036118, Csmd3, 9630028H03Rik, Gm12296, Srrm2, Gm41505, Opcml, Chst9, Gm20319, A230004M16Rik, Ahi1, Nrg3os, Gm42418, AC149090.1* |

**Table D. WGCNA module preservation statistics**. Modules defined in the nucleus-level networks were evaluated in the within-mouse metacell data (4,303 metacells from 64,569 nuclei, k = 15) using the permutation-calibrated statistics of Langfelder et al. (2011). Z summary is the mean of the median density and median connectivity Z-scores. Strong, Z > 10; Moderate, 2 ≤ Z ≤ 10; Not preserved, Z < 2. Modules CA1-1 and CA3-1, from which the hub gene analysis derives, are shown in bold.

| **Cell type** | **Module** | **Genes** | **Z density** | **Z connectivity** | **Z summary** |
| --- | --- | --- | --- | --- | --- |
| CA1 | CA1-12 | 269 | 71.43 | 4.58 | 38.00 |
|  | **CA1-1** | **409** | **65.58** | **6.12** | **35.85** |
|  | CA1-10 | 70 | 41.11 | -2.40 | 19.35 |
|  | CA1-5 | 103 | 30.68 | 1.61 | 16.14 |
|  | CA1-13 | 126 | 29.58 | -0.83 | 14.37 |
|  | CA1-14 | 72 | 25.57 | 2.23 | 13.90 |
|  | CA1-7 | 101 | 25.19 | 1.68 | 13.43 |
|  | CA1-8 | 99 | 27.32 | -3.40 | 11.96 |
|  | CA1-3 | 3,664 | 14.03 | 2.45 | 8.24 |
|  | CA1-9 | 78 | 15.46 | 0.62 | 8.04 |
|  | CA1-15 | 97 | 12.49 | -3.20 | 4.65 |
|  | CA1-2 | 975 | 6.41 | 2.73 | 4.57 |
|  | CA1-11 | 88 | 10.99 | -3.21 | 3.89 |
|  | CA1-6 | 15,225 | -0.48 | -0.30 | -0.39 |
|  | CA1-4 | 634 | -8.90 | -3.61 | -6.25 |
| CA3 | CA3-38 | 61 | 84.72 | 2.32 | 43.52 |
|  | CA3-41 | 114 | 69.86 | 2.25 | 36.06 |
|  | CA3-19 | 86 | 68.48 | 2.63 | 35.56 |
|  | CA3-5 | 65 | 66.36 | 2.06 | 34.21 |
|  | CA3-22 | 78 | 69.66 | -5.71 | 31.97 |
|  | CA3-9 | 306 | 56.88 | 7.04 | 31.96 |
|  | CA3-31 | 50 | 61.22 | 2.07 | 31.64 |
|  | CA3-32 | 90 | 60.53 | 2.58 | 31.56 |
|  | CA3-23 | 57 | 56.19 | 2.60 | 29.40 |
|  | **CA3-1** | **325** | **43.93** | **7.23** | **25.58** |
|  | CA3-27 | 120 | 42.73 | 4.43 | 23.58 |
|  | CA3-10 | 227 | 41.55 | 5.17 | 23.36 |
|  | CA3-8 | 94 | 46.89 | -2.38 | 22.25 |
|  | CA3-12 | 155 | 45.65 | -4.13 | 20.76 |
|  | CA3-14 | 76 | 34.36 | 4.36 | 19.36 |
|  | CA3-13 | 129 | 42.05 | -4.07 | 18.99 |
|  | CA3-26 | 85 | 32.52 | -3.82 | 14.35 |
|  | CA3-36 | 81 | 25.72 | 1.57 | 13.65 |
|  | CA3-30 | 88 | 19.14 | 4.32 | 11.73 |
|  | CA3-3 | 84 | 19.09 | 3.24 | 11.17 |
|  | CA3-16 | 1,921 | 11.94 | 5.31 | 8.63 |
|  | CA3-21 | 1,369 | 7.66 | 6.89 | 7.28 |
|  | CA3-29 | 98 | 9.33 | 3.92 | 6.63 |
|  | CA3-25 | 63 | 7.92 | 3.11 | 5.51 |
|  | CA3-33 | 84 | 5.10 | 5.52 | 5.31 |
|  | CA3-35 | 64 | 6.61 | 3.97 | 5.29 |
|  | CA3-39 | 68 | 5.25 | 3.52 | 4.39 |
|  | CA3-24 | 115 | 3.73 | 4.98 | 4.36 |
|  | CA3-17 | 73 | 4.05 | 4.11 | 4.08 |
|  | CA3-40 | 109 | 6.32 | 0.32 | 3.32 |
|  | CA3-28 | 88 | 1.32 | 3.86 | 2.59 |
|  | CA3-18 | 2,021 | 1.73 | 3.25 | 2.49 |
|  | CA3-6 | 3,806 | 5.52 | -0.65 | 2.43 |
|  | CA3-2 | 6,220 | 3.85 | -0.57 | 1.64 |
|  | CA3-20 | 89 | 3.31 | -1.74 | 0.79 |
|  | CA3-15 | 78 | -2.27 | 2.87 | 0.30 |
|  | CA3-34 | 86 | 0.67 | -1.10 | -0.21 |
|  | CA3-37 | 63 | 1.01 | -2.71 | -0.85 |
|  | CA3-11 | 282 | -8.75 | -3.88 | -6.31 |
| DG | DG-12 | 13,302 | 4.61 | -0.30 | 2.16 |
|  | DG-1 | 10,804 | -6.21 | -2.04 | -4.13 |
| Inhibitory | Inhibitory-22 | 1,531 | 70.03 | 3.59 | 36.81 |
|  | Inhibitory-11 | 560 | 47.01 | 1.40 | 24.21 |
|  | Inhibitory-18 | 1,120 | 40.05 | 3.75 | 21.90 |
|  | Inhibitory-14 | 1,183 | 31.84 | 3.70 | 17.77 |
|  | Inhibitory-2 | 1,071 | 25.46 | 3.46 | 14.46 |
|  | Inhibitory-4 | 428 | 21.78 | 4.33 | 13.06 |
|  | Inhibitory-6 | 2,475 | 16.31 | 0.56 | 8.44 |
|  | Inhibitory-7 | 223 | 0.80 | 2.69 | 1.74 |
|  | Inhibitory-3 | 229 | 4.86 | -2.47 | 1.20 |
|  | Inhibitory-12 | 492 | -1.51 | -1.04 | -1.28 |
|  | Inhibitory-13 | 253 | -5.54 | -1.37 | -3.46 |
|  | Inhibitory-1 | 12,762 | -5.10 | -2.86 | -3.98 |
| Astro | Astro-2 | 471 | 141.11 | 4.46 | 72.78 |
|  | Astro-8 | 490 | 126.83 | 4.84 | 65.84 |
|  | Astro-11 | 364 | 100.20 | 4.33 | 52.26 |
|  | Astro-6 | 428 | 64.19 | 3.15 | 33.67 |
|  | Astro-9 | 173 | 62.49 | 3.73 | 33.11 |
|  | Astro-15 | 166 | 47.92 | 3.69 | 25.80 |
|  | Astro-5 | 440 | 34.37 | 2.99 | 18.68 |
|  | Astro-13 | 445 | 23.94 | 5.58 | 14.76 |
|  | Astro-10 | 240 | 16.90 | 4.33 | 10.61 |
|  | Astro-7 | 496 | 12.02 | 4.48 | 8.25 |
|  | Astro-12 | 113 | 7.35 | 3.79 | 5.57 |
|  | Astro-4 | 868 | 3.60 | 3.92 | 3.76 |
|  | Astro-14 | 125 | 1.83 | 3.72 | 2.77 |
|  | Astro-3 | 1,056 | 0.40 | 2.73 | 1.57 |
|  | Astro-1 | 15,645 | 0.67 | 0.04 | 0.36 |
| Oligo | Oligo-2 | 2,096 | 49.74 | 0.84 | 25.29 |
|  | Oligo-8 | 336 | 42.83 | 0.61 | 21.72 |
|  | Oligo-12 | 6,173 | 14.24 | 1.62 | 7.93 |
|  | Oligo-3 | 170 | -1.66 | -4.21 | -2.93 |
|  | Oligo-4 | 938 | -3.13 | -6.70 | -4.91 |
|  | Oligo-1 | 12,817 | -5.92 | -7.89 | -6.90 |
|  | Oligo-9 | 597 | -5.09 | -9.29 | -7.19 |
| Microglia | Microglia-2 | 2,588 | 31.42 | 1.93 | 16.68 |
|  | Microglia-18 | 185 | 30.33 | 1.50 | 15.92 |
|  | Microglia-13 | 243 | 28.04 | 1.57 | 14.80 |
|  | Microglia-3 | 583 | 20.70 | 2.61 | 11.66 |
|  | Microglia-10 | 145 | 17.14 | 1.97 | 9.56 |
|  | Microglia-12 | 1,765 | 7.88 | 2.54 | 5.21 |
|  | Microglia-4 | 299 | 8.56 | 0.29 | 4.42 |
|  | Microglia-20 | 160 | 5.84 | 2.54 | 4.19 |
|  | Microglia-1 | 4,210 | 4.84 | 3.41 | 4.12 |
|  | Microglia-14 | 83 | 5.16 | 1.89 | 3.53 |
|  | Microglia-16 | 146 | 4.79 | 1.64 | 3.22 |
|  | Microglia-15 | 249 | 5.99 | -0.14 | 2.93 |
|  | Microglia-6 | 246 | 4.10 | 1.49 | 2.79 |
|  | Microglia-7 | 201 | 3.79 | 0.74 | 2.26 |
|  | Microglia-11 | 227 | 2.71 | 1.67 | 2.19 |
|  | Microglia-8 | 730 | 2.12 | 1.73 | 1.92 |
|  | Microglia-19 | 135 | 0.76 | 2.07 | 1.42 |
|  | Microglia-5 | 304 | 0.92 | 0.13 | 0.52 |
|  | Microglia-9 | 7,549 | 3.13 | -2.22 | 0.45 |
|  | Microglia-17 | 687 | -5.85 | -2.18 | -4.01 |

85 of 110 modules (77%) were preserved; 49 (45%) showed strong preservation.

III. **SUPPLEMENTARY INFORMATION**

**A. Module-level organization of the excitotoxic hippocampal transcriptome: potentially relevant implications.** The following summaries are hypothesis-generating interpretations of module composition and enrichment results, not demonstrated mechanisms.

| 1 | Module-level analysis identified modules whose members span translational control, mitochondrial metabolism, and synaptic regulation across hippocampal populations. |
| --- | --- |
| 2 | Under KA3, Cd47 KO mice showed larger changes in module–trait correlation than their vehicle counterparts (more negative Δr, **Fig 3B**). The modules with the largest changes were, in descending order, DG-1, Inhibitory-1, Oligo-1, CA3-1, CA1-1 and Astro-1. Of these, CA1-1, CA3-1, DG-1 and Microglia-9 share a high proportion of their gene membership (**Fig 4**), and their gene set is enriched for ribosomal biogenesis, translation elongation and oxidative phosphorylation terms (**Figs 5**, **K–M**). We describe this as a translational–bioenergetic gene set common to these modules; we did not measure translational or mitochondrial activity and do not infer it. The overlap between neuronal and microglial modules indicates shared gene membership, not shared function. |
| 3 | Under KA25, fewer modules showed a substantial change in correlation in Cd47 KO mice; those with the largest changes were, in descending order, DG-12, DG-1, Inhibitory-1, Oligo-1 and CA1-1 (**Fig 3B**). The synaptic modules DG-1 and DG-12 showed the largest negative Δr, indicating that co-expression among transcripts encoding vesicle components and neurotransmitter-release proteins is least stable under the convulsive dose. A genotype-associated signal arises from CA3-2, which is enriched for non-motile cilium assembly and shifted its eigengene association with treatment in WT but not in Cd47 KO mice. This raises the testable hypothesis that CD47 is required for a ciliary transcriptional response to excitotoxic challenge; we did not image cilia, quantify ciliary protein or manipulate ciliary signaling, and the observation rests on a single module in a single cell type. Together these observations describe three groupings: a translational–bioenergetic gene set, synaptic modules whose co-expression is least stable at the convulsive dose, and a genotype-associated ciliary module. |
| 4 | Beyond the shared modules, cell-type-specific networks show distinct enrichment patterns. Across excitatory neurons (CA1, CA3, DG), modules CA1-3, CA1-7, CA3-9 and CA3-16 return three groups of enrichment terms: synaptic signaling and plasticity; stress-associated transcriptional regulation; and structural and metabolic terms. The first comprises postsynaptic density, NMDA receptor, neurexin/neuroligin and PLC-β terms. The second comprises AP-2/MAPK and calcium-regulated transcriptional terms, most prominently in CA1-7, together with lipid metabolism terms. The third, in CA3-9, comprises focal adhesion, PI3K–Akt–mTOR, extracellular matrix, collagen biosynthesis and Netrin-1 terms. These are enrichment results describing module composition; no functional consequence is demonstrated. |
| 5 | Inhibitory interneuron modules return three groups of enrichment terms. Inhibitory-2, -14 and -18 are enriched for postsynaptic density and adhesion terms associated with GABAergic transmission. Inhibitory-6 and -7 are enriched for Golgi–ER transport, ubiquitination, insulin signaling and mitochondrial iron–sulfur biogenesis terms. Inhibitory-11 and -22 are enriched for Rho/Rac1, axon guidance and ribosomal biogenesis terms. Inhibitory-6, -18 and -22 are the three modules that reach mouse-level significance for the treatment term (see main text). |
| 6 | Glial modules return immune- and metabolism-annotated enrichment terms. Astrocyte modules are enriched for Rho GTPase, cytoskeletal, proliferative and DNA-repair terms, and for fatty-acid β-oxidation and glutamate transport terms. Microglial modules are enriched for PI3K–Akt–mTOR and adhesion terms (Microglia-2/4), proliferation terms (Microglia-5/6), and ribosomal and mitochondrial terms (Microglia-9). Microglia-12 is enriched for neurexin/neuroligin terms. Oligo-2 is enriched for Rho/CDC42 and cytoskeletal terms rather than myelin biosynthesis terms. Microglial modules are the least well preserved of any cell type (**Supplementary Table 4**) and are interpreted with correspondingly lower confidence. |
| 7 | Across populations, the enrichment terms returned differ by cell type: excitatory neuron modules return synaptic and extracellular matrix terms, inhibitory neuron modules return metabolic and trafficking terms, and glial modules return immune and metabolic terms. Differences in module–trait correlation between genotypes are described in the main text as changes in co-expression structure; whether they correspond to differences in expression level was tested separately at the level of the mouse. |
| 8 | The genes with the highest and most consistent degree centrality across neuronal modules were Fth1, Rplp1 and Apoe. Their annotated functions fall into three areas: iron sequestration and oxidative defense, translational control, and lipid transport. High co-expression centrality does not establish that these genes are functionally important or that they regulate the modules in which they sit; they are presented as candidates for functional follow-up. |

**B. Module Characterization**

To aid interpretation of the glial WGCNA results, the enrichment terms associated with astrocyte, microglial, and oligodendrocyte modules are summarized below. For each module the table gives a potential biological interpretation, the enriched pathways or terms returned, a potentially associated cellular feature, and a potential biological implication. These are hypothesis-generating annotations of module composition, not demonstrated functions.
